# Investigating Molecular Exciton Polaritons Using *Ab Initio* Cavity Quantum Electrodynamics

**DOI:** 10.1021/acs.jpclett.3c01294

**Published:** 2023-06-21

**Authors:** Braden M. Weight, Todd D. Krauss, Pengfei Huo

**Affiliations:** † Department of Physics and Astronomy, University of Rochester, Rochester, New York 14627, United States; ‡ Department of Chemistry, University of Rochester, Rochester, New York 14627, United States; § The Institute of Optics, Hajim School of Engineering, University of Rochester, Rochester, New York 14627, United States

## Abstract

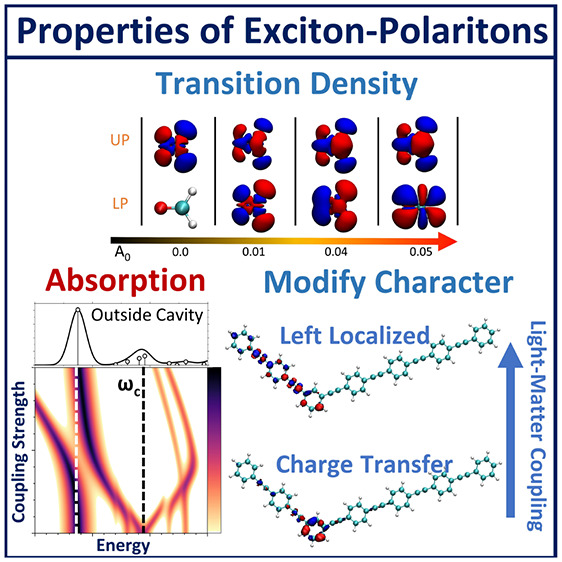

Coupling molecules to the quantized radiation field inside an optical cavity creates a set of new photon–matter hybrid states called polariton states. We combine electronic structure theory with quantum electrodynamics (QED) to investigate molecular polaritons using *ab initio* simulations. This framework joins unperturbed electronic adiabatic states with the Fock state basis to compute the eigenstates of the QED Hamiltonian. The key feature of this “parametrized QED” approach is that it provides the exact molecule–cavity interactions, limited by only approximations made in the electronic structure. Using time-dependent density functional theory, we demonstrated comparable accuracy with QED coupled cluster benchmark results for predicting potential energy surfaces in the ground and excited states and showed selected applications to light-harvesting and light-emitting materials. We anticipate that this framework will provide a set of general and powerful tools that enable direct *ab initio* simulation of exciton polaritons in molecule–cavity hybrid systems.

Coupling molecules to the quantized radiation field inside an optical cavity creates a set of new photon–matter hybrid states, called polariton states.^1−3^ These polariton states have delocalized excitations among coupled molecules and the cavity mode, which have been shown to facilitate new chemical reactivities.^1,3,4^ Theoretical investigations play a crucial role in understanding new principles in this emerging field and have suggested interesting reaction mechanisms enabled by cavity quantum electrodynamics (QED).^5−38^ Polariton chemistry thus provides a potentially new strategy for controlling chemical reactivity in a general way by tuning the fundamental properties of photons and provides a new paradigm for enabling chemical transformations that can profoundly impact several fields of chemistry, including catalysis and energy production.

In recent work, traditional electronic structure methods have been generalized to include the effects of quantum light–matter interactions and used to determine the polaritonic states of molecule–cavity hybrid systems. These efforts include cavity QED density functional theory (QED-DFT),^20,23,39,40^ time-dependent DFT (QED-TD-DFT),^20,22,41−43^ coupled cluster (QED-CC) and its equation-of-motion extension (QED-EOM-CC),^24,39,44^ or full configuration interaction (FCI) methods.^39,45^ In this way, the cavity photonic degrees of freedom (DOFs) and the molecular electronic DOFs are treated on the same quantum mechanical footing, where the total polariton wave function is expressed as a linear combination of different configurations (e.g., Slater determinants of single-particle states), with each configuration composed as a tensor product of the electronic configuration and the photonic configuration. These methods will be termed self-consistent quantum electrodynamic (scQED) methods. As is well-known in these many-body theories, the correlation between electrons captured by these methods varies widely due to the intrinsic approximations used in each method. These approximations force the electron–photon correlation to be treated with a level of approximation (or worse) similar to that of the electron–electron correlation. In particular, for the scQED-DFT approaches, when a less accurate exchange-correlation functional is used to describe the electron–photon coupling, the accuracy of the calculation is drastically reduced.^46^

An alternative approach is to solve the same problem in two steps. This procedure requires one to obtain the electronic adiabatic states using existing electronic structure methods, followed by constructing the total light–matter Hamiltonian using these adiabatic states for the electronic DOFs and Fock states for the photonic DOFs. Then, one directly diagonalizes the total light–matter Hamiltonian to obtain polariton states.^47−52^ For this procedure, only the molecular energies and dipole operator matrix elements are required as input in the dipole-gauge Pauli–Fierz (PF) Hamiltonian under the long-wavelength approximation (see eq 1). In this work, this procedure is termed the parametrized QED (pQED) scheme.

In principle, both pQED and scQED yield identical results under the complete basis limit. Compared to scQED, the pQED scheme is much simpler in the sense that it does not require additional redevelopment of electronic structure theory for the QED Hamiltonian as well as the simplicity that comes with a non-self-consistent solution (through direct diagonalization). In addition, as we mentioned above, pQED has the exact electron–phonon interaction (correlation), whereas some scQED approaches, for example, scQED-DFT, must approximate such electron–photon correlation,^46^ which can lead to inaccurate results. On the contrary, it is important to note that the scQED schemes may require substantially less computational effort than the analogous pQED scheme, on which we will elaborate in the conclusion. Despite the enormous progress in both scQED and pQED schemes, what is generally missing is a consistent comparison of both approaches and assessment of the strengths and limitations of each method under different scenarios. In particular, an open question in the field of *ab initio* polariton chemistry is whether a pQED calculation can provide the same level of accuracy as a scQED simulation.^42,53,54^

In this paper, we use the pQED approach to compute molecular exciton–polariton properties and directly compare them to the existing work based on the scQED approaches. We will directly assess the accuracy of the pQED approach by computing the eigenspectrum of the polaritonic system. In addition, we also present new theoretical metrics for analyzing the excited state properties of the molecule–cavity hybrid systems, which will be a valuable tool for understanding how forming polaritons can influence the ground and excited state properties of the molecule. Finally, we will investigate how molecule–cavity interactions can influence the fundamental property of molecules, including dramatically changing the excited state charge transfer character of the 3–5-poly phenylene ethynylene (35PPE) molecule and computing the polaritonic absorption spectra of a coupled single-walled carbon nanotube (SWCNT) that contains ∼1000 carbon atoms with an optical cavity.

We use the PF Hamiltonian^40,55−59^ to describe the interactions between *ab initio* molecular systems and the photon field. The PF Hamiltonian^58,59^ is expressed as
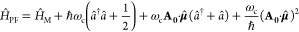
1where *Ĥ*_M_ is the bare molecular Hamiltonian,  is the Hamiltonian of the bare cavity photon field (under the single-mode assumption), ω_c_**A**_**0**_·**μ̂**(*â*^†^ + *â*) is the molecular cavity coupling term under the dipole (length) gauge, and  is the dipole self-energy (DSE), which is essential for the correct description of a bounded ground state^55^ as well as for including other effects for energy and off-diagonal coupling corrections at large light–matter coupling strengths.

The total dipole operator of the molecule is **μ̂** = ∑_*i*_*z*_*i*_**R̂**_*i*_ – ∑_*k*_**r̂**_*k*_, where **R̂**_*i*_ is the position operator of nucleus *i*, with charge *z*_*i*_, and **r̂**_*k*_ is the position operator of electron *k* (with the unit negative charge). In addition, ω_c_ is the frequency of the mode in the cavity, *â*^†^ and *â* are the photonic creation and annihilation operators, respectively, and  and  are the photonic coordinate and momentum operators, respectively.

For a FP cavity, the coupling strength is **A**_0_ = *A*_0_**ê**, where **ê** is the unit vector of the field polarization and  is the field intensity, where  is the quantization volume inside the cavity and *ε*_0_ is the permittivity. Throughout this Letter, we treat *A*_0_ (in atomic units) as a parameter. This parameter is related to the commonly used Jaynes–Cummings (JC) Hamiltonian coupling strength *g*_c_, where *g*_c_ = ω_c_*A*_0_μ_01_. In the standard JC Hamiltonian, only two electronic states with a single dipole matrix element μ_01_ exist.

The molecular Hamiltonian (in the position representation) is expressed as *Ĥ*_M_ = *T̂*_**R**_ + *Ĥ*_el_(**r**, **R**), where *T̂*_**R**_ = – ∑_*i*_ℏ^2^∇_**R**_*i*__^2^/2*M*_*i*_ is the nuclear kinetic energy operator and *Ĥ*_el_(**r**, **R**) = *T̂*_**r**_ + *V̂*_coul_(**r**, **R**) is the electronic Hamiltonian, with electronic kinetic energy *T̂*_**r**_ and Coulomb potential *V̂*_coul_(**R**, **r**) among electrons and nuclei. The electronic adiabatic state |ψ_α_(**R**)⟩ is defined as the eigenstate of *Ĥ*_el_ as
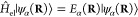
2where α = 0, 1, 2 ..., and |ψ_0_(**R**)⟩ is the ground electronic state of the matter. The matrix elements of the total dipole operators can be obtained using the adiabatic states as
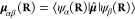
3

In a similar sense of defining the electronic Hamiltonian and corresponding eigenvalue equation for the matter, one can define the polaritonic Hamiltonian^22,40,42,53,60^ as *Ĥ*_pl_ ≡ *Ĥ*_PF_ – *T̂*_**R**_, which includes all operators of the molecules and cavity, except for nuclear kinetic energy operator *T̂*_**R**_. As such, the polariton state is defined as the eigenstate of *Ĥ*_pl_ through the following eigenvalue problem
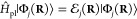
4where *Ĥ*_pl_ ≡ *Ĥ*_PF_ – *T̂*_**R**_ is the polariton Hamiltonian, |Φ_*j*_(**R**)⟩ is the polariton eigenstate, and  is the polariton potential energy surface. As one can clearly see, both |Φ_*j*_(**R**)⟩ and  parametrically depend on nuclear configuration **R**. We denote |Φ_0_(**R**)⟩ as the ground state of *Ĥ*_pl_.

To solve the eigenvalue problem in eq 4, one can represent the polaritonic state through the convenient adiabatic–Fock basis as
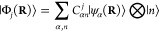
5where *C*_*αn*_^*j*^ is the expansion coefficient of the *j*th polariton for the *αn*th basis state, |ψ_α_(**R**)⟩ is the αth electronic adiabatic state (i.e., the eigenstate of *Ĥ*_el_ in eq 2), and |*n*⟩ is the Fock state, i.e., the eigenstate of . At a particular molecular geometry, direct diagonalization of the polaritonic matrix, with matrix elements defined as ⟨ψ_β_, *m*|*Ĥ*_pl_|ψ_α_, *n*⟩, provides both expansion coefficients *C*_*αn*_^*j*^ = ⟨ψ_α_(**R**), *n*|Φ_*j*_(**R**)⟩ and the polariton energies . With the pQED approach, we treat the number of electronic and photonic basis states as convergence parameters^42^ for the polaritonic properties, e.g., the convergence of the lowest few eigen-energies  (see a convergence test in Figure S1). For practical use, one can further truncate the electronic basis to a reasonable number while obtaining identical chemical insight with the dominating error stemming from the choice of the electronic structure method (to be discussed in Figure 2).

It is worth noting that in the community of *ab initio* QED for realistic molecular systems, often a coherent state transformation^44,56,61^ is performed on the PF Hamiltonian (eq 1). This unitary-transformed PF Hamiltonian can be written solely in terms of the fluctuations in the dipole operator *Δμ̂* = μ̂ – ⟨μ̂⟩ and its square (*Δμ̂*)^2^. In this sense, one can shift away the direct coupling term by choosing a basis of shifted (and electronic state specific) Fock states for the photonic DOFs. Many works in scQED approaches use this shifted basis, which is often termed the generalized coherent state (GCS) basis^44,62^ and is closely related to the polarized Fock state (PFS) basis.^59,61^ The GCS basis is convenient for self-consistent methods because the expected value of the dipole operator can be evaluated at each self-consistent cycle and achieve the optimal photonic basis at each iteration. The GCS photonic basis states can be interpreted as coherent states (i.e., linear combinations of vacuum Fock states), which, in general, are expected to provide a more rapidly converging basis. However, in this Letter, we use the unperturbed (unshifted) vacuum Fock states for the sake of simplicity, because the primary complication resides in the truncation of the electronic basis rather than the photonic one for realistic, *ab initio* systems.^42^

In addition, all polaritonic observables in this Letter between the ground and the *j*th polariton state are conveniently computed as
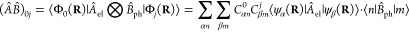
6where *Â*_el_ and *B̂*_ph_ are operators in the electronic and photonic Hilbert subspaces, respectively, {|ψ_α_(**R**)⟩, |ψ_β_(**R**)⟩} are electronic adiabatic states, {|*n*⟩, |*m*⟩} are photonic Fock states, and {*C*_*αn*_^*j*^} are the expansion coefficients in eq 5 in the adiabatic–Fock basis. For example, *A*_*αβ*_ could be the molecular dipole matrix, transition density, or natural transition orbitals, while *B*_*nm*_ could be the photon number matrix or photonic transition density. See Methods for more information about the computation of the individual electronic and photonic properties. Overall, the pQED approach provides a convenient and straightforward procedure for the computation of polaritonic properties, because the approach leverages the extensive, thoroughly tested, and widely available methods developed in the electronic structure community to solve eq 2 in an accurate and efficient fashion. The pQED approach does not require any redevelopment of the new electronic–photonic structure theory but rather utilizes what is already available to the community.

Figure 1 presents the results of molecular polaritons generated by coupling the excited electronic states of the formaldehyde molecule with a single-mode cavity for various cavity frequencies ω_c_ and coupling strengths *A*_0_. This system has been recently explored in ref (22) using the scQED-TD-DFT scheme. Here, we use the pQED-TD-DFT procedure to obtain the polariton PES and transition density (Computational Details) for the molecule–cavity hybrid systems. All of the results obtained in pQED are in agreement with those obtained using scQED-TD-DFT.^22^

**Figure 1:**
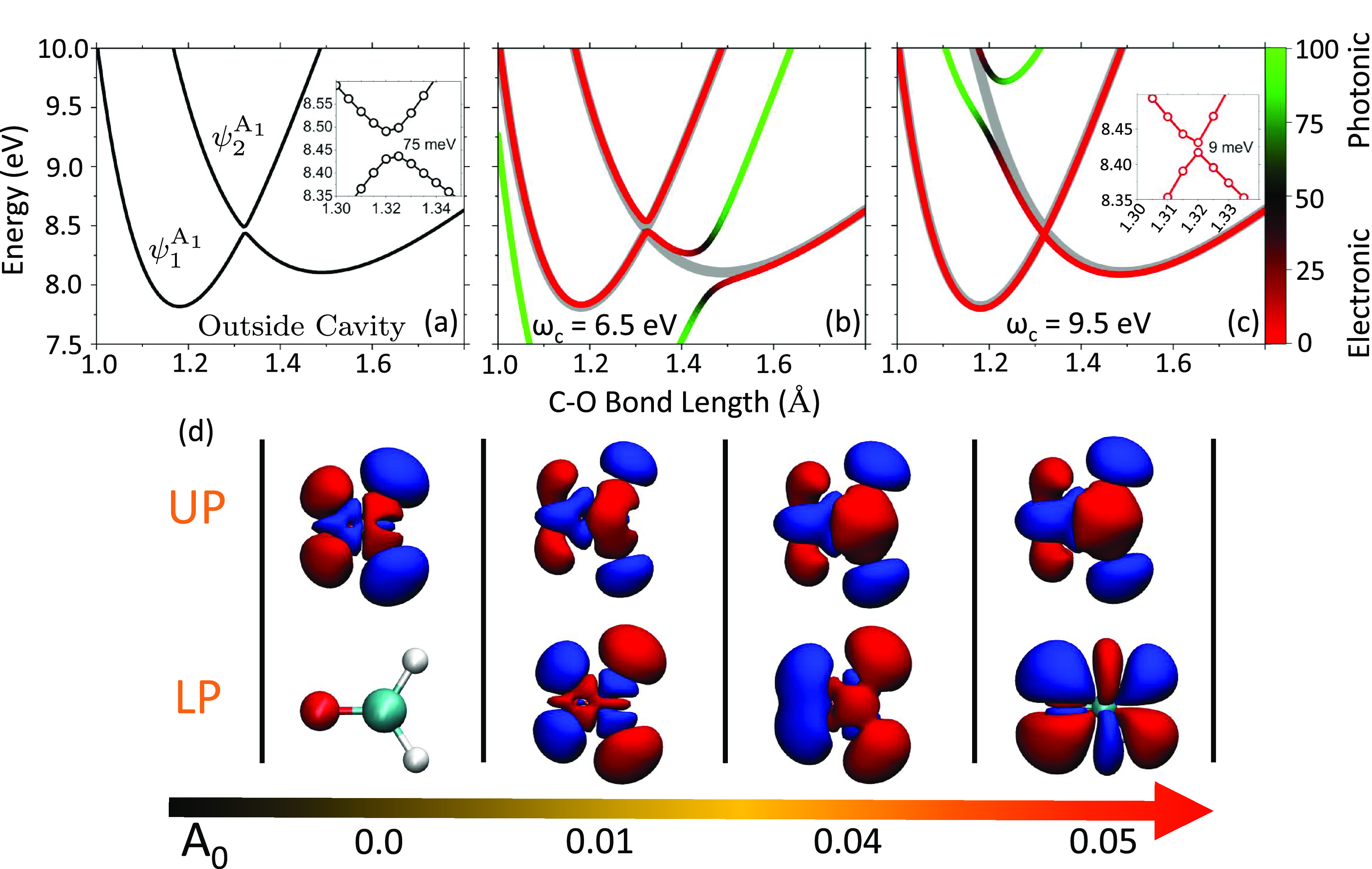
(a) Adiabatic potential energy surface of the formaldehyde molecule’s *A*_1_-symmetry excited states as a function of C–O bond length. The inset presents the avoided crossing between the two adiabatic states. (b and c) Polariton excited state energy  of the formaldehyde–cavity hybrid system, with coupling strength *A*_0_ = 0.04 au and cavity frequencies ω_c_ = 6.5 eV and ω_*c*_ = 9.5 eV, respectively. Cavity polarization **ê** is parallel to the C–O bond. For panels b and c, the color map indicates the photonic character, and the cavity-free electronic states are shown as thick gray lines. The inset in panel c shows the reduction of the avoided crossing from 75 to 9 meV. (d) Transition density ρ_0*j*_^M^(**r**) (see eq 10) of the upper (UP) and lower (LP) polaritons for *A*_0_ = 0, 0.01, 0.04, and 0.05 au at ω_c_ = 7.92 eV with a C–O bond length of 1.22 Å.

Figure 1a presents the two lowest-energy ^1^*A*_1_-symmetry excited states of the formaldehyde molecule (adiabatic states of *Ĥ*_el_) as a function of the C–O bond length, denoted as |ψ_1_^*A*_1_^⟩ and |ψ_2_^*A*_1_^⟩. The C–O bond length was scanned by changing only the location of the oxygen atom, keeping all other nuclei frozen. At a C–O bond length of 1.22 Å, these two states correspond to |ψ_4_(**R**)⟩ and |ψ_7_(**R**)⟩ but vary in adiabatic label along the C–O coordinate. The symmetry (i.e., *A*^1^) of the adiabatic state determines the orientation of the primary, non-zero, ground-to-excited transition dipole. Note that there is an avoided crossing near a C–O bond length of 1.3–1.4 Å, with an energy gap of ∼75 meV (see the inset) at the LR-TD-DFT level of theory.

Panels b and c of Figure 1 present the polaritonic PESs  (defined in eq 4) of the hybrid system with field polarization direction **ê** along the C–O bond and cavity photon frequencies of ℏω_c_ = 6.5 eV (Figure 1b) and ℏω_c_ = 9.5 eV (Figure 1c), respectively. The character of the polaritonic excited states is color-coded (see the color bar on the right of panel c) according to the average photon number ⟨Φ_*j*_|*â*^†^*â*|Φ_*j*_⟩ in the cavity. In Figure 1b, the green curve is largely composed of the photon-dressed ground state |ψ_0_(**R**)⟩⊗|1⟩ ≡ |ψ_0_(**R**), 1⟩. Near a C–O bond length of 1.45 Å, the |ψ_0_(**R**)⟩⊗|1⟩ state crosses the |ψ_1_^*A*_1_^(**R**)⟩⊗|0⟩ state, which is an excited electronic state with zero photons (thick gray curve in Figure 1b). The light–matter interaction (in eq 1) hybridizes the two states and generates the polaritonic states, with the energy splitting generated commonly termed the Rabi splitting. At this nuclear configuration, both polariton states contain roughly equal contributions of the excitonic and photonic character (indicated as 50% on the color bar with the color black). In addition, the molecule–cavity interaction produces additional hybridization among electronic states with the mixed ground and excited adiabatic character as well as photonic excitation character. The original adiabatic potential energy minimum located near a C–O bond length of 1.5 Å for the lower-energy ψ_1_^*A*_1_^ state (see panel a) has now been removed (see panel b). The new lowest-energy excited state polariton has a totally downward slope toward the same minimum location as the ground polariton state |Φ_0_(**R**)⟩, with nearly 100% photonic character (green color) in that region.

Figure 1c presents similar features of the polariton potential with a cavity frequency of ℏω_c_ = 9.5 eV. For this case, the photon-dressed ground state |ψ_0_(**R**)⟩⊗|1⟩ (green curve) hybridizes with |ψ_2_^*A*_1_^⟩⊗|0⟩ (gray) and generates a large Rabi splitting at *R* = 1.25 Å. Interestingly, due to the light–matter interaction, the energy of the middle polariton curve (red curve in *R* = 1.35 Å) in this panel is lower than that of the original adiabatic state |ψ_2_^*A*_1_^,0⟩ (gray). The inset shows that the avoided crossing has been reduced to 9 meV, a reduction of 1 order of magnitude compared to the original avoided crossing in the bare molecule (Figure 1a). This is because the photon-dressed ground state (green) “pushes” down upon the ψ_2_^*A*_1_^ state (due to light–matter coupling), effectively reducing the magnitude of the avoided crossing. In the Supporting Information (Additional Results of the Systems in the Main text), we present the magnitude of the avoided crossing as a smooth function of the cavity energy in Figure S3b. An important feature of this change in the avoided crossing is that the character of both states involved is retained compared to that outside the cavity (i.e., mainly electronic excitation), where both states exhibit negligible amounts of photonic contributions. Direct control over the relative energy of electronic states while maintaining their original character is a useful concept and design principle in processes controlled by non-adiabatic coupling between the excited electronic states.

Figure 1d presents the real-space projected transition density (eq 10), where the photonic DOFs have been traced out, leaving only the electronic contributions. The light–matter hybridization leads to superpositions between photon-dressed electronic states, which leads to various transition densities that have been mixed through the polaritonic expansion coefficients in the adiabatic–Fock basis (see eq 5). The changes in the polaritonic transition density are presented as a function of coupling strength *A*_0_ (varied along the horizontal axis of panel d) for the upper and lower polaritons, with a C–O bond length of 1.22 Å and at cavity energy ω_c_ = 7.92 eV. Under this configuration, the cavity is nearly resonant with the molecular adiabatic transition from the ground state to the ψ_1_^*A*_1_^ state at the Franck–Condon points. Through the mixing of the characters of electronic states, the transition density is modified for each coupling strength. The transition density results obtained from the pQED simulation presented in panel d are visually identical to those obtained from scQED (at the level of TD-DFT) in ref (22).

Figure 2 presents a cavity-mediated proton transfer reaction by coupling the aminopropenal molecule to the cavity. This system was recently investigated in ref (46) using scQED-HF, scQED-DFT, and scQED-CC to examine the ground state barrier and product energies of the proton transfer reaction. This asymmetric reaction is an ideal example of assessing the accuracy of the corresponding pQED calculations. Several theoretical works have demonstrated that the ground state of a molecular system can be significantly modified by coupling to a cavity photon mode with a photon frequency in the electronic excitation range.^18,23,41,44,45,56,63,64^ These modifications are induced by indirect couplings between different photon-dressed states, due to the presence of both transition and permanent dipoles^59^ as well as directly through the DSE. For example, the |*g*, 0⟩ state couples with |*g*, 1⟩ through ⟨*g*, 1|**μ̂**(*â*^†^ + *â*)|*g*, 0⟩ = **μ**_*gg*_⟨1|(*â*^†^ + *â*)|0⟩, and |*g*, 1⟩ couples to |*e*, 0⟩ through **μ**_*ge*_⟨1|(*â*^†^ + *â*)|0⟩. As such, the |*g*, 0⟩ and |*e*, 0⟩ states are indirectly coupled to each other (through the light–matter interactions), and the ground state properties can also be significantly influenced under the strong light–matter interaction coupling strength. In addition, the DSE term (see eq 1 and the paragraph below) could also significantly influence the ground state properties and reactivities, as demonstrated in ref (46), and the contribution of DSE becomes more important in the strong and ultrastrong coupling regime.^35,55,58,59^ This is because any ground-to-excited state matrix elements of the DSE operator, *Ĥ*_DSE_ (last term in eq 1), can be expressed as ⟨ψ_0_|*Ĥ*_DSE_|ψ_α_⟩ = ω_c_*A*_0_^2^∑_β_μ_0β_μ_*βα*_, where α and β are any of the electronic adiabatic states. One can clearly see that the DSE term can connect electronic adiabatic states far apart in energy. An intuitive and simple understanding of cavity modification of the molecular ground state is provided with a new representation of the cavity Fock states, termed the polarized Fock states.^59,61^

**Figure 2:**
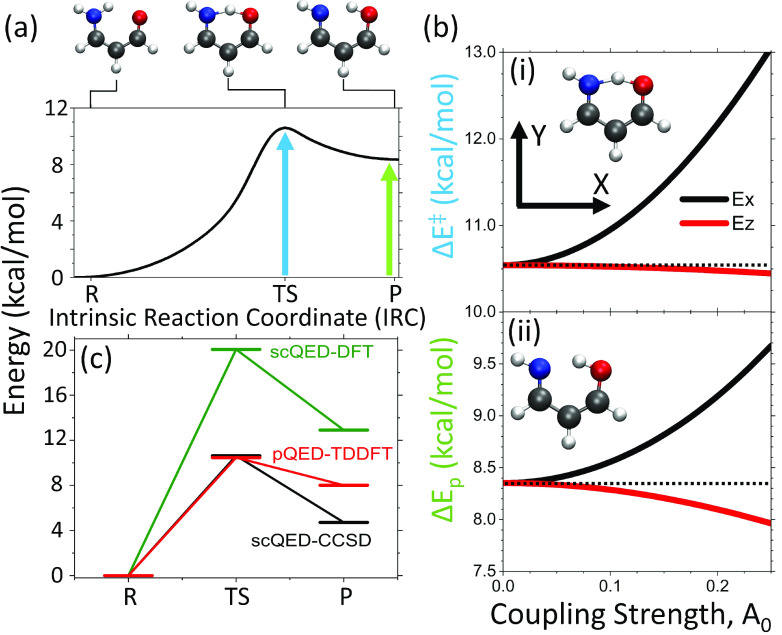
(a) Potential energy surface (PES) of the aminopropenal proton transfer reaction. (b) Energy of the (i) transition state (TS) and (ii) product (P) state as functions of coupling strength *A*_0_, with cavity frequency ω_c_ = 3.0 eV and the field polarization direction along the *X* (solid black line) and *Z* (solid red line) directions. (c) Relative energy between the transition state and the reactant and between the product and the reactant, with an *X*-polarized cavity field. The results are obtained using pQED-TD-DFT/ωB97XD (red), whereas the results of scQED-DFT/OEP (green) and scQED-CCSD (black) are taken from ref (46). The results obtained from scQED-CCSD are used as a more accurate benchmark.

Figure 2a presents the potential energy surface (PES) of the reaction outside the cavity, computed using intrinsic reaction coordinate (IRC) analysis at the level of density functional theory (DFT) (see Methods). The reaction has a potential energy barrier of ∼10 kcal/mol from the reactant (R) to the transition state (TS). This is in nearly perfect agreement with the coupled cluster (CC) results from ref (46). However, the energy difference between the reactant and product from the DFT calculation (∼8 kcal/mol) is much larger than the CC results (5 kcal/mol), due to the limitations of DFT. Following the previous work,^46^ we define the *X* and *Y* directions of the molecule, indicated in the inset of Figure 2a. The *Z* direction is perpendicular to the defined *X–Y* plane. Cavity polarization direction **ê** will be aligned with the *X* or *Z* direction of the molecule.

Figure 2b(i) presents the TS barrier height  (polariton energy difference between the transition state and reactant nuclear configuration) on polariton ground state |Φ_0_(**R**)⟩ as a function of light–matter coupling *A*_0_ (arbitrary units) with the cavity frequency set to ω_c_ = 3.0 eV. The optimized reactant, transition state (TS), and product geometries were calculated outside the cavity, as was done in ref (46). Note that the mechanism for modifying the ground state potential energy surface in this case is due to the coupling to the excited states (through indirect couplings and through DSE as mentioned above), which is different from the related vibrational strong coupling modification for the ground state reactivities.^1,4−6^ The *X* polarization (solid black line) demonstrates a stronger effect on barrier height because a strong transition dipole exists between the ground state and second electronic excited state (μ_01_^*x*^ ∼ 1.47/1.26 au) as well as strong ground state permanent dipole (μ_00_^*x*^ ∼ 1.37/0.90 au) in the reactant/transition state geometries (see Figure S2 for dipole matrices for aminopropenal at the reactant, transition state, and product geometries, including the 20 lowest-energy adiabatic states). The magnitude of the ground-to-excited state transition dipole in the *Z* direction is almost negligible in comparison, showing an only slight decrease in the barrier height (solid red line).

Figure 2b(ii) depicts the effects on product energy  on polariton ground state |Φ_0_(**R**)⟩ as a function of coupling strength *A*_0_ with the same cavity frequency ω_c_ = 3.0 eV. The effects of the cavity for the product energy are reduced for the *X* polarization in comparison to the TS and increased for the *Z* polarization. Similar to the case for the TS, the *X* polarization presents a continuous increase in energy while the *Z* polarization shows a slight decrease. The influence of the *Z*-polarized cavity on *ΔE*^⧧^ [panel b(i)] and *ΔE*_p_ [panel b(ii)] obtained from the pQED-TD-DFT calculation is also consistent with the previous work^46^ using the scQED-CC approach. In particular, with the *Z* polarization and coupling strength of *A*_0_ = 0.22 au, the difference between the prediction of *ΔE*^⧧^ obtained from the pQED-TD-DFT and scQED-CC methods is <10 meV. For *ΔE*_p_, the difference between the two methods is <5 meV. This further exemplifies that the pQED-TD-DFT method and the scQED-CC methods provide the same quantitative description for the prediction of *ΔE*_p_ and *ΔE*^⧧^, without the additional effort of developing new DFT functionals.

For a direct comparison to self-consistent methods, Figure 2c showcases the scQED-DFT/OEP,^46^ scQED-CCSD,^46^ and pQED-TD-DFT/ωB97XD methods in calculating the transition state (TS) and product (P) ground state reaction points. We assume high-level scQED-CCSD as the benchmark. The scQED-DFT/OEP method predicts a much higher barrier and product energies relative to those of the reactant by roughly ∼8 kcal/mol in comparison to the scQED-CCSD result. This inaccuracy is likely due to the approximate electron–photon correlation assumed in the density functional used in the scQED-DFT/OEP approach.^46^ The pQED-TD-DFT/ωB97XD approach from the work presented here, on the contrary, provides nearly perfect agreement with the scQED-CCSD transition state while exhibiting an overestimation of the product energy by ∼4 kcal/mol. Overall, the pQED-TD-DFT/ωB97XD approach outperforms the scQED-DFT/OEP approach in both the transition state and product geometries compared with the benchmark scQED-CCSD results.

It should be noted that the level of DFT used in this work was at the hybrid ωB97XD level while in ref (46) a new functional was used that includes approximate electron–photon exchange termed the optimized effective potential (OEP) approximation.^23^ The differences in the cavity effects between scQED-CCSD and the method used in this work are <30 meV for the transition state geometry, which is well within the degree of accuracy between DFT and CC levels of theory for these systems. The higher accuracy achieved by the pQED approach is likely due to two primary effects: (I) the exact electron–photon correlation provided by the direct diagonalization of the exact interaction term used in eq 1 and (II) the heightened level of theory for the bare electron–electron correlations due to the more accurate ωB97XD hybrid functional. As such, despite using the TDDFT level theory for the molecule, the results of the pQED approach can achieve the level of accuracy provided by scQED-CCSD, and they outperform the scQED-DFT.

Next, we move toward computing the polaritonic properties in the excited state for interesting chemical and physical processes. Figure 3 presents the polariton natural transition orbital (NTO) calculations of coupling the 35 PPE molecules (conjugated polymer) to an optical cavity. For a detailed explanation of the polaritonic NTOs, see Theoretical Details of the Transition Density in the Supporting Information. In particular, we aim to demonstrate how to manipulate the character and energetic alignment of an excited charge transfer state (which is optically inactive) via coupling a nearby excited state with a cavity mode.

**Figure 3:**
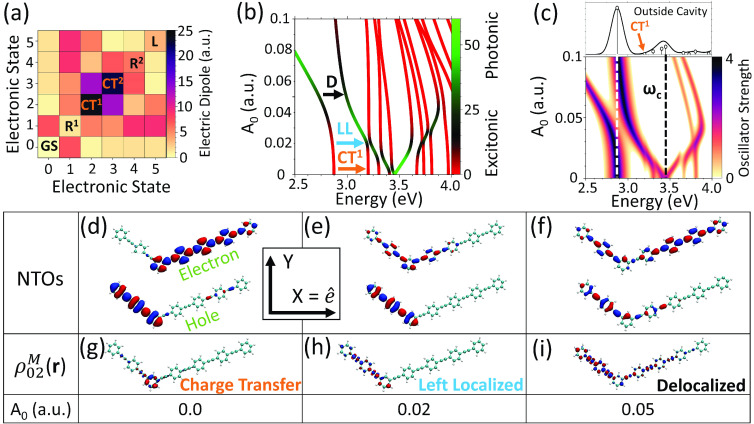
Tuning excited state polariton properties by coupling a 35PPE molecule to an optical cavity with a ω_c_ of 3.45 eV. (a) Dipole matrix of the 35PPE molecule (outside the cavity), with labels indicating the character of each excitation along the permanent dipole. These states are the ground state (GS), ππ* transition localized to the right arm (R^1^), charge transfer state 1 (CT^1^), charge transfer state 2 (CT^2^), ππ* right-localized (R^2^), and ππ* left-localized (L). The color bar indicates the magnitude of the dipole matrix element. (b) Polariton energy spectrum as a function of coupling strength *A*_0_. The color of the curves denotes the percent of the photonic character of the state, with excitonic (red) or photonic character (green). (c) Absorption spectrum outside the cavity (top) and excitonic absorption spectra of the 35PPE molecule coupled to the cavity (bottom) as a function of coupling strength *A*_0_. The white dashed vertical line indicates the transition energy to the bare molecular ψ_1_ state, and the black dashed vertical line indicates the cavity frequency, which is in resonance with the ψ_1_ → ψ_5_ transition energy. (d–f) Natural transition orbitals (NTOs) for the ground-to-second state polaritonic excitation for three different coupling strengths (*A*_0_ = 0.0, 0.02, and 0.05 au). (g–i) Matter-projected polaritonic transition density ρ_02_^M^ for the same coupling strength as shown in panels d–f, respectively. At zero coupling strength, the second excited polaritonic transition corresponds to the CT^1^ state.

Figure 3a presents the transition dipole matrix in the bare molecular system, where 0 labels the ground state and 5 labels the fifth excited state. Along the diagonal (permanent dipole elements), the character of each transition is labeled, where R (L) indicates a ππ* excitation localized on the long right (short left) arm and CT indicates a charge transfer state between arms. The NTOs outside the cavity are shown in Figure S4 for the lowest six molecular excitations. In many photovoltaic or light emission applications, one aims to control the energetic positioning of the charge transfer states with respect to other types of excitations, e.g., ππ*, to provide the most beneficial pathway for the excited state population transfer. Here, we demonstrate the ability to control the energetic alignment and character of a low-energy charge transfer state (CT^1^) via coupling a higher-energy electronic transition (L) with a non-zero oscillator strength to the cavity.

Figure 3b presents the polaritonic energy as a function of coupling strength *A*_0_, where the color bar indicates the character of the states (photonic as green and excitonic as red). Here, the cavity frequency is in resonance with the fifth molecular excitation (denoted as the L state in Figure 3a) at ω_c_ = 3.45 eV with cavity polarization in the *X* direction (see the inset between panels d and e). Figure 3c shows the excitonic absorption spectra (see Methods) as a function of coupling strength *A*_0_, where the vertical black dotted line (at 3.45 eV) indicates the cavity frequency (in resonance with the higher-energy ψ_0_ → ψ_5_) and the vertical white dotted line (at 2.8 eV) indicates the low-energy ψ_0_ → ψ_1_ transition. In the panel above Figure 3c, the absorption spectrum of the bare 35PPE molecule outside the cavity is shown. To analyze the character of the low-energy CT^1^ excitation as a function of coupling strength *A*_0_, we compute the polaritonic NTOs (Figure 3d–f) and transition density (Figure 3g–i) at three coupling strengths *A*_0_ = 0.0, 0.02, and 0.05 au (see Methods and Theoretical Details of the Transition Density in the Supporting Information for more details on NTO calculations).

At *A*_0_ = 0.0 au, the character of the CT^1^ exciton is evident as the electron and hole NTOs are spatially separated across both arms, while the transition density is present only at the corner where the electron/hole NTOs are strongly overlapping. At low values of coupling strength (for *A*_0_ < 0.02 au), there are a series of crossings between polaritonic states that exhibit a variety of splittings on the order of ≲20 meV. Although most ground-to-excited matter states have a very small oscillator strength, all states are effectively coupled by the transition dipole matrix (Figure 3a). The polaritonic states exchange character as a function of light–matter coupling strength *A*_0_. Near the coupling strength *A*_0_ = 0.02 au, the character of the, previously unperturbed, second polaritonic state (originally CT^1^ at *A*_0_ = 0.0 au) becomes mixed with the descending-in-energy state largely composed of the original ψ_5_ exciton (L defined in Figure 3a), which exhibited a ππ* excitation localized to the left arm. At *A*_0_ = 0.02 au, the CT^1^ state and the descending polaritonic state are maximally mixed, which localizes the transition density (Figure 3h) to the left arm. The polariton NTOs (Figure 3e) show that the hole is largely unperturbed by this mixing due to the shared hole character between the original CT^1^ and the L excitons (see Figure 3a and Figure S5 for the transition dipole matrix for the 6 and 20 lowest-energy electronic states). The electron distribution is more drastically affected, moving from completely right-localized to mostly left-localized with some non-zero right-arm character near the corner. Additionally, the magnitudes of both hole and electron NTOs are reduced due to the non-zero mixture of the cavity photon, which is most prominent for the electron.

At a larger light–matter coupling strength (*A*_0_ = 0.05 au), the lower polariton branch (which is descending in energy as the coupling strength increases) starts to mix with the lowest-energy excited state of the bare molecule (originally R character at *A*_0_ = 0.0 au), which adds right-localized character to the Φ_2_ state (originally CT^1^ at *A*_0_ = 0.0 au) through off-resonant direct (although weak due to the small transition dipole) mixing between |*g*⟩⊗|1⟩ and |CT^1^⟩⊗|0⟩ as well as through direct dipole self-energy (DSE) term μ̂^2^ (see eq 1) between the |CT^2^⟩⊗|0⟩ and |S_5_⟩⊗|0⟩ states. This effectively delocalizes the transition density across both arms. The NTOs in panel f demonstrate these modifications more clearly. The excited electron becomes mostly right-localized; however, the excited hole retains much of its left-localized character while simultaneously decreasing in magnitude due to the larger contribution of the photonic character after mixing.

The results of the NTOs explain the features present in the transition density, showing the electron distribution is delocalized while the hole distribution is only weakly delocalized at this coupling strength. As an additional analysis technique, the fragmented polaritonic transition density matrix can be found for the |Φ_2_⟩ polaritonic excitation in Figure S6a–c at three values of coupling strength, which provides more information regarding the spatial coherence for each polaritonic excitation.^65^

As noted above, the NTOs for the electron and hole distributions for the 35PPE molecule exhibit asymmetric changes upon coupling to the cavity, such that the electronic NTO was largely modified while the hole NTO was only weakly modified. The presence of asymmetric modifications implies an asymmetry of affected occupied and virtual orbitals that comprise the various bare molecular excitations. In practical terms, for the cavity to induce large changes in the electron’s NTO distribution and not the hole’s, the cavity-induced changes to electronic states would need to mix (or cause crossings between) excited states that share a common dominant virtual orbital in its expansion. For example, let us consider an excitation primarily composed of the *j* → *a* single-particle transition (i.e., in a singly excited Slater determinant sense) and another, *j* → *b*. Because the two excitations share the same occupied orbital (i.e., *j*), the cavity-induced mixing would primarily affect the excited electron distribution (because *a* ≠ *b*) and not the hole distribution. We expect this to be the reason for the asymmetric changes in the electron and hole NTOs present in Figure 3. Such an asymmetry in the cavity-induced changes to molecular properties (in occupied/virtual orbitals in ref (56) or electron/hole NTOs in this work) has been demonstrated in previous work.^56^ However, the investigation of the single-particle orbitals and their role in the transition are beyond the scope of this work and will be the subject of future work.

In the example provided in Figure 3, we are able to effectively manipulate the optically inactive CT^1^ state in the bare molecular system by coupling to a higher-energy electronic transition with non-zero oscillator strength. The character as well as the energetic alignment of the second polaritonic state with respect to nearby bare molecular excited states was modulated by tuning light–matter coupling strength *A*_0_. These results indicate a route toward the experimental tunability of CT systems that can be utilized in realistic light-harvesting or light-emitting systems to achieve a high degree of exciton splitting or radiative recombination, respectively, after photoexcitation processes.

Figure 4 presents the results of a single-walled carbon nanotube (SWCNT) system (∼1000 atoms) coupled to an optical cavity, which has been the subject of recent experimental interest in molecule–cavity coupling.^66−71^ Semiconducting SWCNTs are known to have weak photoluminescence due to low-lying optically inactive states below the so-called band-edge *E*_11_ exciton, which is the 6th excited state, and we denote |ψ_6_⟩ ≡ |*E*_11_⟩. Figure 4a provides the transition dipole matrix for the pristine (6,5) SWCNT system, showing a sparse matrix with only the ground-to-E_11_ (i.e., the sixth bare molecular transition) transition to be optically allowed with a large dipole moment of ∼28 au, and small dipole matrix elements between other excited states. The dipole matrix of CNT is thus akin to the two-level system studied in quantum optics. This is in strong contrast to previous molecules studied in this work (see Figure 3a and Figures S2, S3, and S5), where many states are connected with each other through transition dipoles.

**Figure 4:**
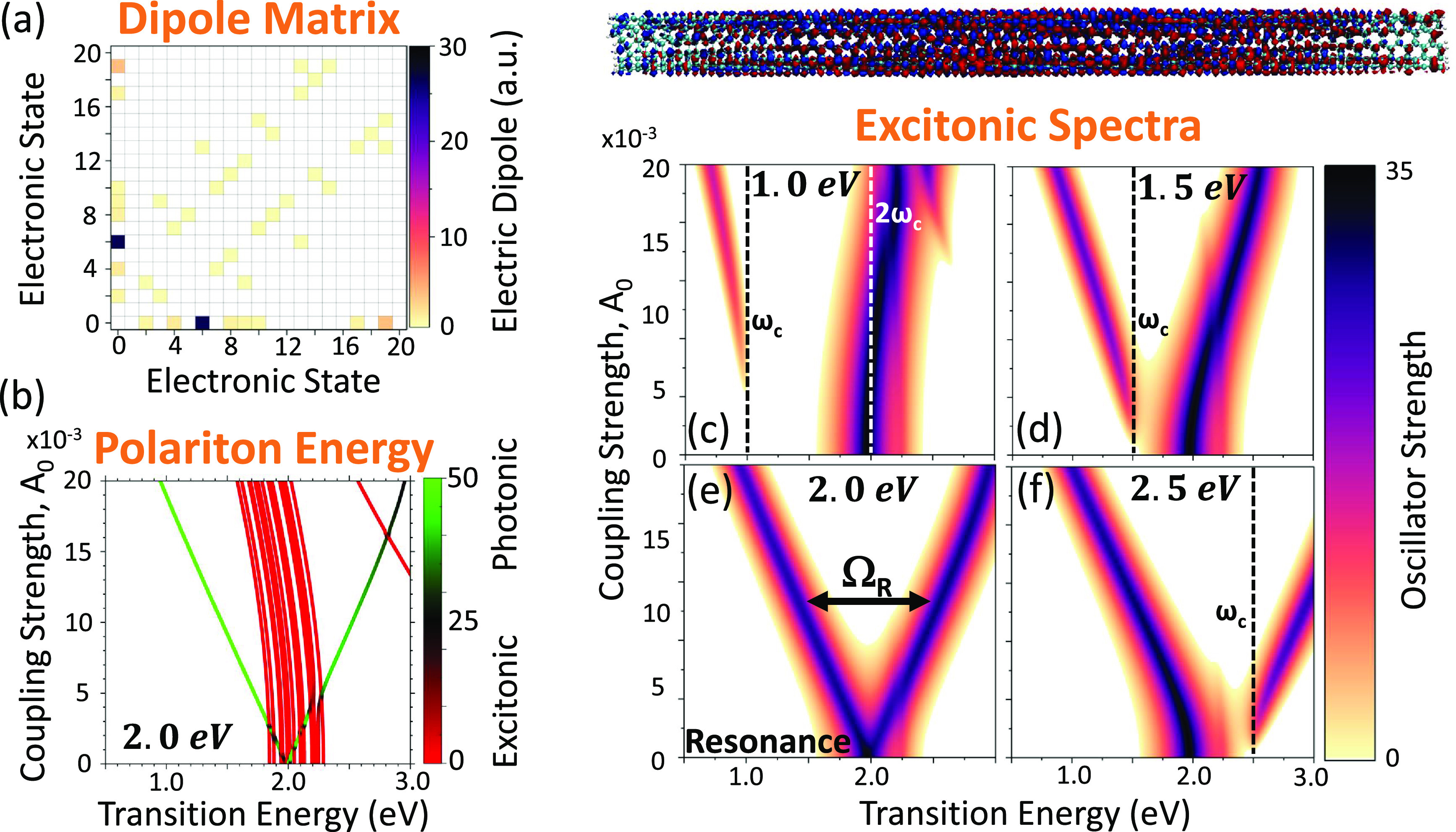
(a) Electronic transition dipole matrix for the pristine (6,5) single-walled carbon nanotube (SWCNT). The color bar indicates the magnitude of the dipole matrix element. (b) Polaritonic energy as a function of coupling strength *A*_0_, where the cavity frequency is in resonance with the bright *E*_11_ matter excitation (with ω_c_ = 2.0 eV). The color bar indicates the percent of photonic character. (c–f) Excitonic absorption spectra plotted as a function of the transition energy and coupling strength *A*_0_. The Lorentzian energy-broadening parameter is σ = 0.1 eV. The cavity frequency (ω_c_) is taken to be (c) 1.0 eV (half-resonance), (d) 1.5 eV, (e) 2.0 eV (resonant with bright molecular transition, *E*_11_), and (f) 2.5 eV. The polaritonic transition density at *A*_0_ = 0.0 au is shown above the spectra for the ground-to-bright *E*_11_ state transition.

The LR-TD-DFT level of theory used in this work predicts that the ground to E_11_ state transition has an energy just below 2.0 eV, which agrees with previous theoretical calculations.^72^ The top right panel (above Figure 4c,d) provides the real-space projected bare electronic transition density of the ground-to-E_11_ state exciton, demonstrating its delocalized excitation character. Semiconducting SWCNTs, when functionalized with covalent adducts forming a hybridization defect, exhibit bright emission features due to the addition of a defect-associated exciton below the band edge, which have been the subject of much experimental and theoretical work over the past decade for their use as single-photon light sources and for their tunable emission at low-energy telecommunications wavelengths.^72−90^ The simplicity of this system’s dipole matrix (akin to a two-level system) will lead to clear theoretical predictions and a new pathway toward the tunable manipulation of SWCNTs by coupling to optical cavities, without the need for chemical functionalization.

Figure 4b shows the polaritonic energy as a function of the coupling strength for the SWCNT–cavity hybrid system when the cavity is in resonance with the bright *E*_11_ transition. The characters of the polariton states are also indicated on the curve, with red as exciton character and green as photon character. A majority of states centered around 2.0 eV have excitonic character (red), showcasing the many optically inactive (weakly dipole-coupled) molecular states housed in the same region of energy. In addition, one notices the nearly symmetric Rabi splitting due to the |ψ_0_⟩⊗|1⟩ and |*E*_11_⟩⊗|0⟩ hybridization, with decoupled nearby states [due to the sparsity of the dipole matrix (Figure 4a)] that act as spectator states, until an extremely large coupling of *A*_0_ ∼ 0.02 au, where all states begin to exhibit a decrease in energy.

Figure 4c shows the excitonic spectra of the system inside the cavity (see Methods) when the cavity frequency is half of the transition energy to the bright |*E*_11_⟩ state. Here, the |ψ_0_,2⟩ photon-dressed ground state is in resonance with the bright |*E*_11_⟩ state. There is a weak repulsion of the effective upper and lower polaritons that arises from the weak coupling between the |ψ_0_⟩⊗|1⟩ and |*E*_11_⟩⊗|0⟩ states. At very large coupling strengths, the lowest-energy excited polaritonic state |Φ_1_⟩ becomes partially bright due to mixing with the *E*_11_ state over the large energy difference of nearly 1.0 eV. Panels d–f of Figure 4 show the same information but with a varied cavity frequency: (d) ω_c_ = 1.5 eV, (e) ω_c_ = 2.0 eV, and (f) ω_c_ = 2.5 eV. Interestingly, the resonant case (panel e) shows nearly perfect Rabi splitting (denoted as Ω_R_) as a function of coupling strength. The negative detuning (panel d) and positive detuning (panel f) also demonstrate an interesting behavior in which the bright character of the *E*_11_ state can be manipulated by tuning the cavity frequency and coupling strength by either (d) blue-shifting or (f) red-shifting the bright state. In the latter, the polaritonic system should be extremely emissive in the infrared telecommunications wavelengths. Until now, chemical functionalization^80,83,91^ was one of the only methods^92,93^ for brightening the emission of these materials, and our results demonstrate another avenue toward this goal through molecular cavity QED.

In this Letter, we use the rigorous PF QED Hamiltonian^58,94^ to describe the molecule–cavity interactions and use adiabatic electronic structure information along with the Fock states of the cavity mode as the fundamental building blocks to compute polariton eigenstates. We refer to this approach as the pQED approach. Using the time-dependent density functional theory (TD-DFT) as the electronic structure method, we demonstrated the accuracy of the pQED for predicting polariton excited state potential as well as excited state properties, such as the polariton transition density (Figure 1). These results are consistent with the scQED-DFT work.^22^ We further assess the accuracy and performance of pQED by computing the ground state proton transfer reaction. The effects of the cavity on the ground state are through indirect light–matter couplings, as well as the dipole self-energy term. The results from the pQED approach with the TD-DDFT electronic structure method quantitatively agree with those obtained using scQED-CC and are better than the results from scQED-DFT.^46^ This is likely due to the exact light–matter coupling Hamiltonian used in pQED and the approximate electron–photon correlation functional used in scQED-DFT.^46^

We further used the pQED approach to tune the energetic alignment and electronic character of the charge transfer states in the 35PPE molecule by resonant coupling to a higher-energy bright molecular excited state. As a function of the light–matter coupling strength, the character of the higher-energy bright exciton was mixed with the character of the charge transfer state, changing its properties, namely, exciton localization. To illustrate these changes, we introduced the polaritonic natural transition orbitals, transition density, and transition density matrix analysis methods, which are standard quantum chemical tools for analyzing excited electronic states.

We also discovered a tunability of the state character and energetic alignment of charge transfer states (Figure 3), which can be achieved through coupling the photonic transition to a non-charge transfer state that has large transition dipole moments. These bright states can further influence the charge transfer state via excited-to-excited state transition dipole moments appearing in the dipole self-energy term μ̂^2^ and eventually through the off-resonant light–matter interaction term μ̂(*â*^†^ + *â*), effectively mixing the bright character with the charge transfer character in a tunable way. The basic mechanism is similar to a previously proposed model of polariton-mediated charge transfer (see Figure 7 of ref (35)). To illustrate these changes, we introduced the polaritonic natural transition orbital, transition density, and transition density matrix analysis methods, which are standard quantum chemical tools for analyzing excited electronic states.

Finally, we applied the pQED scheme to investigate the polaritonic properties of a single-walled carbon nanotube, a system of recent experimental interest for polaritonic applications.^66,67,70,71,83,87,95^ We showed that through light–matter coupling one can red-shift the bright *E*_11_ character of the tube below the band of dark states (which makes these materials dark to emission outside the cavity), thus enabling strong optical emission from these materials inside the cavity without the need for chemical functionalization.

It is important to note that the scQED schemes may require substantially less computational effort than the analogous pQED scheme. For computing the ground polaritonic state in particular (Figure 2), the pQED scheme requires knowledge of the excited electronic states to converge the ground polaritonic state, while the cost of the scQED scheme is roughly the same as that of a standard ground state calculation due to the variational approach in the basis of single-particle orbitals rather than many-body excited states (see ref (37)). The calculations of excited polaritonic states in both schemes are more similar in terms of computational expense, because both require an approximate excited state method (e.g., TD-DFT, EOM-CC, etc.). The pQED scheme may require the calculation of many more (∼10–20) bare electronic excitations to converge the few lowest-energy polaritonic states, whereas the scQED scheme requires only the calculation up to the number of polaritonic states needed. Note that even in the excited state, the convergence in the number of included virtual orbitals in the scQED scheme is still required, especially at large light–matter coupling strengths. As such, we believe that the self-consistent evaluation of the PF Hamiltonian will be a much more general and reliable scheme for producing results converging toward chemical accuracy, especially at very strong light–matter coupling strengths. However, we propose the pQED scheme as a useful and valuable tool aimed at the convenient calculation of polaritonic properties for application-style studies, where only semiquantitative trends may be important.

Overall, we demonstrated the accuracy and usage of the pQED approach as a conceptually simple and easy-to-implement method. Future directions will be focused on a consistent comparison of both pQED and scQED approaches and an assessment of their strengths and limitations in various numerical and chemical situations.

## Methods

The transition density operator for the ground to *j*th state in the polaritonic system ρ̂_0*j*_ can be written as
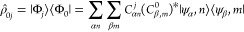
7where each polaritonic state has been expanded in the basis of molecular and photonic states (see eq 5). We are interested in examining the changes in the electronic part of the system as a result of hybridization, so we first trace out the photonic degrees of freedom and define the following molecular-projected transition density operator
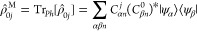
8where we have explicitly used the orthonormality relation of the Fock states ⟨*n*|*m*⟩ = δ_*nm*_. This expression implies that ρ̂_0*j*_^M^ mixes all molecular transition densities according to the expansion coefficients of the polaritonic states. One can perform an integration over all but one of the electronic DOFs, arriving at matter-projected one-particle transition density matrix ρ_0*j*_^M^(**r**_e_, **r**_h_) as follows
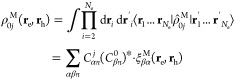
9where ξ_*βα*_^M^(**r**_e_, **r**_h_) is the bare molecular single-particle transition density. The diagonal elements of ρ_0*j*_^M^(**r**_e_, **r**_h_) comprise the real-space projected transition density of the polariton system
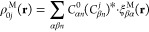
10where ξ_*βα*_^M^(**r**) ≡ ρ_0*j*_^M^(**r**, **r**) = ψ_α_(**r**). One can also define a real-space transition density matrix by examining the spatial coherence between a single electronic coordinate and a photonic coordinate, with additional discussion provided in Theoretical Details of the Transition Density in the Supporting Information.

Figures 3 and 4 show the excitonic absorption spectra computed using the following expression^53^
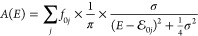
11where  is the transition energy between the ground and *j*th polaritonic states, σ = 0.1 eV is the broadening parameter, and *f*_0*j*_ is the oscillator strength between the ground and *j*th polaritonic state
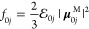
12where **μ**_0*j*_^M^ is the molecular part of the polaritonic dipole calculated from eq 6 with . The matrix elements of **μ̂** are computed according to eq 3.

All molecules were optimized with DFT in the ground state electronic configuration, without the influence of the cavity. Formaldehyde, 3–5-poly phenylene ethynylene (35PPE) and H_2_ utilized the B3LYP/6-311++G** level of theory while aminopropenal was calculated with the ωB97XD/6-311++G** level of theory. The excited states were calculated with linear-response time-dependent DFT (LR-TD-DFT) at the same level of theory. All DFT, LR-TD-DFT, and natural transition orbital (NTO)^96^ calculations were performed using the Gaussian 16 software package.^97^ All dipole matrix elements, ground-to-excited real-space projected transition densities, and ground-to-excited transition density matrices were computed using Multiwfn version 3.7.^98^ The bare electronic excited-to-excited state transition densities ξ_*βα*_^M^(**r**_e_, **r**_h_) (and subsequently the excited-to-excited state NTOs) can, in principle, be computed via the *Z* vector method^99^ for capturing additional contributions to the various analysis techniques. For the polariton transition density (as well as polariton NTOs) presented in this work, we have not included those electronic excited-to-excited state transition densities, ξ_*βα*_^M^(**r**_e_, **r**_h_). Nevertheless, our results (Figure 1d) are in quantitative agreement with those of scQED-TD-DFT that should have included them implicitly through the self-consistent update of the excited state transition density, suggesting a less important contribution from those electronic transition densities to the ground-to-excited state polaritonic transition densities and NTOs.

For formaldehyde and aminopropenal, we used 500 electronic states and 10 Fock states to solve eq 4. For 35PPE, eq 4 was solved with 100 electronic states and 10 Fock states. The finite-size (6,5) SWCNT was constructed using the visual molecular dynamics (VMD) package^100^ including three unit cells (∼10 nm, 1092 atoms). The edges were properly capped with hydrogens according to previous works.^72,78,79,101−103^ The SWCNT was optimized at the CAM-B3LYP/STO-3G level, followed by the calculation of the 20 lowest-energy vertical excitations at the same level of theory. This functional and basis have been shown to provide the correct excitonic localization and relative energies for these systems.^72−74,78,79,103^ For SWCNT, we used 20 electronic states and five Fock states to solve eq 4. We have carefully checked the convergence with details provided in Convergence test of the pQED approach in the Supporting Information.

For Figure 3, the isovalues for the transition densities and NTOs were chosen to be 0.001 and 0.02, respectively. The absorption spectra were broadened with a Lorentzian distribution with a width σ of 0.1 eV.

## Supporting Information Available

Additional figures that provide additional details regarding the results in the text, a convergence test (with respect to electronic and photonic basis size) for a benzaldehyde molecule as well as additional dipole matrices for all molecules presented in this work, additional plots of the tunability of the avoided crossing in formaldehyde as well as an excited state analysis of the 35PPE molecule inside and outside the cavity, and supporting mathematical derivations and interpretations of the theoretical procedure (PDF)
Transparent Peer Review report available (PDF)
