# Supplementary material for: Investigating Molecular Exciton Polaritons Using Ab Initio Cavity Quantum Electrodynamics
Source: J Phys Chem Lett. 2023 Jun 21;14(25):5901–13. doi: 10.1021/acs.jpclett.3c01294 (PMC10316409; doi:10.1021/acs.jpclett.3c01294)
Supplement: Supplementary file 1 — jz3c01294_si_001.pdf [file jz3c01294_si_001.pdf]

Investigating Molecular Exciton-Polaritons Using Ab Initio Cavity Quantum Electrodynamics

Supporting Information

Braden M. Weight,[*,†] Todd D. Krauss,[‡,¶] and Pengfei Huo[*,‡,¶]

†*Department of Physics and Astronomy, University of Rochester, Rochester, NY 14627, U.S.A.*

‡*Department of Chemistry, University of Rochester, Rochester, NY 14627, U.S.A.*

¶*The Institute of Optics, Hajim School of Engineering, University of Rochester, Rochester, NY 14627, U.S.A.*

E-mail: bweight@ur.rochester.edu; pengfei.huo@rochester.edu

Theoretical Details of the Transition Density.

In order to examine the excited state properties of the polaritonic systems, the one-particle transition density operator[1–3] between the αth and β^{th} molecular states $\hat{\xi}^{\mathrm{M}}_{\alpha\beta}$ is defined as

$$\begin{aligned}\hat{\xi}^{\mathrm{M}}_{\beta\alpha} = |\psi_\alpha\rangle\langle\psi_\beta| &= \int d\mathbf{r}d\mathbf{r}'\psi_\alpha(\mathbf{r})\psi^*_\beta(\mathbf{r}')|\mathbf{r}\rangle\langle\mathbf{r}'| \\ &\equiv \int d\mathbf{r}d\mathbf{r}'\xi^{\mathrm{M}}_{\beta\alpha}(\mathbf{r},\mathbf{r}')|\mathbf{r}\rangle\langle\mathbf{r}'|,\end{aligned} \tag{S1}$$

where $\xi^{\mathrm{M}}_{\beta\alpha}(\mathbf{r},\mathbf{r}') = \psi_\alpha(\mathbf{r})\psi^*_\beta(\mathbf{r}')$, $\psi_\alpha(\mathbf{r}) = \langle\mathbf{r}|\psi_\alpha\rangle$, and $\mathbf{r} = \{\mathbf{r}_1, \cdots, \mathbf{r}_{N_e}\}$, where N_e is the number of electrons. One can perform an integration over all but one of the electronic degrees of freedom (for both the primed and unprimed coordinates) and arrive at the molecular one-

particle transition density matrix $\xi^{\mathrm{M}}_{\beta\alpha}(\mathbf{r}_{\mathrm{e}}, \mathbf{r}_{\mathrm{h}})$, defined as,

$$\xi^{\mathrm{M}}_{\beta\alpha}(\mathbf{r}_{\mathrm{e}}, \mathbf{r}_{\mathrm{h}}) = \int \prod_{i=2}^{N_{\mathrm{e}}} \mathbf{dr}_i \mathbf{dr}'_i \langle \mathbf{r}_1 \cdots \mathbf{r}_{N_{\mathrm{e}}} | \hat{\xi}^{\mathrm{M}}_{\beta\alpha} | \mathbf{r}'_1 \cdots \mathbf{r}'_{N_{\mathrm{e}}} \rangle, \tag{S2}$$

where we have replaced $\mathbf{r}_1$ with $\mathbf{r}_{\mathrm{e}}$ and $\mathbf{r}'_1$ with $\mathbf{r}_{\mathrm{h}}$ on the left-hand side, which helps to signify that one may interpret the two coordinates as the electron and hole position in real space. One can also define the real-space projected transition density for the molecule,

$$\xi^{\mathrm{M}}_{\beta\alpha}(\mathbf{r}) = \int \prod_{i=2}^{N_{\mathrm{e}}} \mathbf{dr}_i \langle \mathbf{r}_1 \cdots \mathbf{r}_{N_{\mathrm{e}}} | \hat{\xi}^{\mathrm{M}}_{\beta\alpha} | \mathbf{r}_1 \cdots \mathbf{r}_{N_{\mathrm{e}}} \rangle, \tag{S3}$$

which is just the diagonal matrix elements of $\xi^{\mathrm{M}}_{\beta\alpha}(\mathbf{r}_{\mathrm{e}}, \mathbf{r}_{\mathrm{h}})$ in Eq. S2. Both $\xi^{\mathrm{M}}_{\beta\alpha}(\mathbf{r}_{\mathrm{e}}, \mathbf{r}_{\mathrm{h}})$ and $\xi^{\mathrm{M}}_{\beta\alpha}(\mathbf{r}_{\mathrm{e}})$ are readily available from excited state electronic structure methods, such as TD-DFT and CIS.

The interpretation of the above transition density matrix $\xi^{\mathrm{M}}_{\beta\alpha}(\mathbf{r}_{\mathrm{e}}, \mathbf{r}_{\mathrm{h}})$ (in the real-space basis) is well-defined.[1] The off-diagonal elements give indication of the spatial coherence between the electron and hole coordinates, indicating the probability of finding an extra electron at $\mathbf{r}_{\mathrm{e}}$ and a hole at $\mathbf{r}_{\mathrm{h}}$ upon transition from adiabtic state ψ_α to ψ_β. The diagonal elements of this matrix can be thought of as a localized excitation at $\mathbf{r}$ upon the electronic transition. This quantity is zero when the electron and hole do no occupy the same $\mathbf{r}$ and can thus be thought of as an electron-hole overlap density. Additionally, in practice one aims to choose a useful representation of the matrix such that the interesting physics is one-dimensional so ξ remains two-dimensional and easily portrayed as a matrix. This can be achieved by binning the three-dimensional real-space grid into fragments (summing all atom-localized atomic orbitals falling within some real-space discretization) or by choosing a basis of atoms (a sum of all atom-localized basis functions on each atom),[1,4] as was done in Fig. 3.

The transition density operator for the ground to j_{th} state in the polaritonic system $\hat{\rho}_{0j}$

can be written as

$$\hat{\rho}_{0j} = |\Phi_j\rangle\langle\Phi_0| = \sum_{\alpha n}\sum_{\beta m} C^j_{\alpha n}(C^0_{\beta,m})^*|\psi_\alpha, n\rangle\langle\psi_\beta, m|, \tag{S4}$$

where each polaritonic state has been expanded in the basis of molecular and photonic states (see Eq. 5). We are interested in examining the changes of the electronic part of the system as a result of hybridization, so we first trace out the photonic degrees of freedom and define the following molecular-projected transition density operator

$$\hat{\rho}^{\mathrm{M}}_{0j} = \mathrm{Tr}_{\mathrm{Ph}}[\hat{\rho}_{0j}] = \sum_{\alpha\beta n} C^j_{\alpha n}(C^0_{\beta n})^*|\psi_\alpha\rangle\langle\psi_\beta|, \tag{S5}$$

where we have explicitly used the orthonormality relation of the Fock states $\langle n|m\rangle = \delta_{nm}$. This expression implies that the $\hat{\rho}^{\mathrm{M}}_{0j}$ mixes all molecular transition densities according to the expansion coefficients of the polaritonic states. Next, we insert two complete sets of position states for all the electronic degrees of freedom such that $\mathbf{r} = \{\mathbf{r}_1, \cdots, \mathbf{r}_{N_\mathrm{e}}\}$ where N_e is the number of electrons in the system.

$$\begin{aligned}
\hat{\rho}^{\mathrm{M}}_{0j} &= \sum_{\alpha\beta n} C^j_{\alpha n}(C^0_{\beta n})^* \int d\mathbf{r}d\mathbf{r}'|\mathbf{r}\rangle\langle\mathbf{r}|\psi_\alpha\rangle\langle\psi_\beta|\mathbf{r}'\rangle\langle\mathbf{r}'| \\
&= \sum_{\alpha\beta n} C^j_{\alpha n}(C^0_{\beta n})^* \int d\mathbf{r}d\mathbf{r}'\psi_\alpha(\mathbf{r})\psi^*_\beta(\mathbf{r}')|\mathbf{r}\rangle\langle\mathbf{r}'| \\
&\equiv \sum_{\alpha\beta n} C^j_{\alpha n}(C^0_{\beta n})^* \int d\mathbf{r}d\mathbf{r}'\xi^{\mathrm{M}}_{\beta\alpha}(\mathbf{r},\mathbf{r}')|\mathbf{r}\rangle\langle\mathbf{r}'|,
\end{aligned} \tag{S6}$$

where $\xi^{\mathrm{M}}_{\beta\alpha}(\mathbf{r},\mathbf{r}') \equiv \psi_\alpha(\mathbf{r})\psi^*_\beta(\mathbf{r}')$ is the bare molecular transition density matrix element including all electronic DOFs. As a note, in the usual electronic structure calculations (Eq. 2) without considering spin-orbit coupling, one can always construct a purely real set of $\{|\psi_\alpha(\mathbf{R})\}\rangle$ in the atomic basis, hence all quantities are completely real. Finally, one can perform an integration over all but one of the electronic DOFs, arriving at the matter-projected one-particle

transition density matrix $\rho_{0j}^{\mathrm{M}}(\mathbf{r}_{\mathrm{e}}, \mathbf{r}_{\mathrm{h}})$ as follows,

$$\begin{aligned}\rho_{0j}^{\mathrm{M}}(\mathbf{r}_{\mathrm{e}}, \mathbf{r}_{\mathrm{h}}) =& \int \prod_{i=2}^{N_{\mathrm{e}}} \mathbf{dr}_i \mathbf{dr}'_i \langle \mathbf{r}_1 \cdots \mathbf{r}_{N_{\mathrm{e}}} | \hat{\rho}_{0j}^{\mathrm{M}} | \mathbf{r}'_1 \cdots \mathbf{r}'_{N_{\mathrm{e}}} \rangle \\ =& \sum_{\alpha\beta n} C_{\alpha n}^{j} (C_{\beta n}^{0})^* \cdot \xi_{\beta\alpha}^{\mathrm{M}}(\mathbf{r}_{\mathrm{e}}, \mathbf{r}_{\mathrm{h}}),\end{aligned} \tag{S7}$$

where $\xi_{\beta\alpha}^{\mathrm{M}}(\mathbf{r}_{\mathrm{e}}, \mathbf{r}_{\mathrm{h}})$ is the bare molecular single-particle transition density matrix (Eq. S2). The diagonal elements of $\rho_{0j}^{\mathrm{M}}(\mathbf{r}_{\mathrm{e}}, \mathbf{r}_{\mathrm{h}})$ comprise the real-space projected transition density of the polariton system,

$$\rho_{0j}^{\mathrm{M}}(\mathbf{r}) = \sum_{\alpha\beta n} C_{\alpha n}^{0} (C_{\beta n}^{j})^* \cdot \xi_{\beta\alpha}^{\mathrm{M}}(\mathbf{r}), \tag{S8}$$

where $\xi_{\beta\alpha}^{\mathrm{M}}(\mathbf{r}) \equiv \psi_\alpha(\mathbf{r})\psi_\beta^*(\mathbf{r})$.

Alternatively, one can use the full polariton transition density operator (Eq. S4)

$$\hat{\rho}_{0j} = \sum_{\alpha\beta, nm} C_{\alpha n}^{0} (C_{\beta n}^{j})^* \int d\mathbf{r} d\mathbf{r}' \xi_{\beta\alpha}^{\mathrm{M}}(\mathbf{r}, \mathbf{r}') |\mathbf{r}, n\rangle \langle \mathbf{r}', m|, \tag{S9}$$

Tracing out, instead, all but one of $\mathbf{r}$ and all $\mathbf{r}'$ through $\int d\mathbf{r}_2 \cdots d\mathbf{r}_{N_{\mathrm{e}}} \cdot \int d\mathbf{r}'_1 \cdots \mathbf{r}'_{N_{\mathrm{e}}}$ for the polariton transition density operator (Eq. S9) one arrives at a similar expression for the polaritonic transition density matrix as follows

$$\rho_{0j}(\mathbf{r}_{\mathrm{e}}, q_{\mathrm{c}}) = \sum_{\alpha n} \sum_{\beta m} C_{\alpha n}^{0} (C_{\beta m}^{j})^* \cdot \xi_{\beta\alpha}^{\mathrm{M}}(r_{\mathrm{e}}) \xi_{mn}^{\mathrm{ph}}(q_{\mathrm{c}}), \tag{S10}$$

where $\xi_{mn}^{\mathrm{ph}}(q_{\mathrm{c}}) \equiv \phi_n(q_{\mathrm{c}})\phi_m^*(q_{\mathrm{c}})$, and $\phi_n(q_{\mathrm{c}}) = \langle q_{\mathrm{c}} | n \rangle$ is the Fock state in the position representation.

One can generalize Eq. S10 to include all possible one-particle matter, photonic, and mixed matter-photonic observables. As such, all polaritonic observables in this letter between the ground and the j_{th} excited polariton state were computed as (equivalently to Eq. 6 in

the main text)

$$
\begin{aligned}
(\hat{A}\hat{B})_{0j} &= \langle \Phi_0 | \hat{A}_{\mathrm{el}} \otimes \hat{B}_{\mathrm{ph}} | \Phi_j \rangle \\
&= \sum_{\alpha n} \sum_{\beta m} (C^0_{\alpha n})^* C^j_{\beta m} \langle \psi_\alpha(\mathbf{R}) | \hat{A}_{\mathrm{el}} | \psi_\beta(\mathbf{R}) \rangle \cdot \langle n | \hat{B}_{\mathrm{ph}} | m \rangle,
\end{aligned}
\tag{S11}
$$

where $\hat{A}_{\mathrm{el}}$ and $\hat{B}_{\mathrm{ph}}$ are operators in the electronic and photonic Hilbert sub-spaces, respectively, $\{|\psi_\alpha(\mathbf{R})\rangle, |\psi_\beta(\mathbf{R})\rangle\}$ are electronic adiabatic states, $\{|n\rangle, |m\rangle\}$ are photonic states, and $C^j_{\alpha n}$ is the expansion coefficient (see Eq. 5). Specifically, $A_{\alpha\beta}$ may be the molecular dipole matrix, transition density, or natural transition orbitals, while B_{nm} may be the photon number matrix, photonic transition density, or photonic dipole matrix. For the NTOs calculation presented in Fig. 3d-f, the electronic NTOs for a given polaritonic transition were constructed by computing the electronic NTOs for the bare molecular system.

Natural transition orbital (NTO)[2,3,5] analysis is another commonly used method to understand the electron and hole distributions in real space, which can be readily evaluated by electronic structure packages through diagonalization (or in general, singular value decomposition) of the transition density matrix, $\xi^{\mathrm{M}}_{\beta\alpha}(\mathbf{r}_{\mathrm{e}}, \mathbf{r}_{\mathrm{h}})$ (see Eq. S1). The dominant singular values $\{s_d\}$ represent the largest contributions to the electron and hole distributions in real-space for a particular $\alpha\beta_{\mathrm{th}}$ electronic transition while retaining the phase information of the orbitals. The transition orbitals are referred to as the highest occupied transition orbital (HOTO) $(\zeta^{\mathrm{H}}_d)^{\mathrm{M}}_{\alpha\beta}$ and lowest unoccupied transition orbital (LUTO) $(\zeta^{\mathrm{L}}_d)^{\mathrm{M}}_{\alpha\beta}$. For the NTOs presented in Fig. 3(c)-(e) of the main text , the polaritonic NTOs $(\Xi^{\mathrm{H/L}}_d)^{\mathrm{M}}_{\alpha\beta}$ for a given polaritonic transition were constructed by computing the electronic NTOs for all bare molecular transitions $(\zeta^{\mathrm{H/L}}_d)^{\mathrm{M}}_{\alpha\beta}$ followed by inserting into Eq. S11 with $\hat{B}_{\mathrm{ph}} = \hat{\mathbb{1}}_{\mathrm{ph}}$, where $\hat{\mathbb{1}}_{\mathrm{ph}}$ is the identity matrix for the photonic DOFs. This will give a similar result as that of Eq. S8 where the photonic DOFs have been traced out, and the examination of the cavity-induced effects on the local electronic structure can be examined. The inclusion of such an analysis of the photonic DOFs are not required due to the simplicity of the single quantized cavity

mode harmonic oscillator structure.

Closely following the notation in Ref. 4, the electronic transition density matrices were constructed using the following relations (noting that we ignore de-excitation coefficients for simplicity),

$$P_{\mu\nu}^{\beta\alpha} = \sum_{o}^{\text{occ}} \sum_{v}^{\text{vir}} \mathcal{W}_{ov}^{\beta\alpha} C_{\mu}^{o} C_{\nu}^{v} \tag{S12}$$

where C_{μ}^{o} corresponds to the expansion coefficient of basis function μ in molecular orbital o and $\mathcal{W}_{o}^{v}$ is the configuration coefficient for the excitation of molecular orbital o to v between electronic states α and β. The $(fg)_{\text{th}}$ element of a fragmented transition density matrix can then be constructed as

$$\xi_{\beta\alpha}^{M}(f,g) = \sqrt{\sum_{\mu\in f} \sum_{\nu\in g} \left(P_{\mu\nu}^{\beta\alpha}\right)^{2}}, \tag{S13}$$

where f and g define the fragments based on the AOs centered on the corresponding fragments in the molecule. This object can then be used in Eq. S11 as $\hat{A}_{\text{el}}$ to construct the matter-projected polaritonic fragmented transition density matrix, as was done in Fig. S6 with 14 fragments.

Theoretical Details of the Absorption Spectra.

In Figs. 3 and 4, the excitonic absorption spectra was computed as,

$$A(E) = \sum_{j} f_{0j} \frac{\frac{\sigma}{\pi}}{(E - E_{0j})^{2} + \frac{\sigma^{2}}{4}}, \tag{S14}$$

where E_{0j} is the transition energy between the ground and j^{th} polaritonic state, $\sigma = 0.1$ eV is the Lorentzian broadening of the energy, and f_{0j} is the oscillator strength between the ground and j^{th} polaritonic state computed as,

$$f_{0j} = \frac{2}{3} E_{0j} |\mu_{0j}^{\text{M}}|^{2}. \tag{S15}$$

Here, μ_{0j}^{M} is the molecular part of the polaritonic dipole calculated from Eq. S11 with $\boldsymbol{\mu}_{0j}^{\mathrm{M}} = \hat{A}\hat{B} = \hat{\boldsymbol{\mu}} \otimes \hat{\mathbb{1}}_{\mathrm{ph}}$ in order to exclude the photonic contribution to the absorption spectra. In principle one could include the photonic contribution; however, the focus of this work was to show changes to the excitonic part of the system.

In principle, another term should be added to account for the photonic part of the absorption/emission, which is proportional to $\hat{A}_{\mathrm{el}} \otimes \hat{B}^{\mathrm{ph}} \sim \hat{\mathbb{1}}_{\mathrm{el}} \otimes \hat{q}_{\mathrm{c}} \sim \hat{\mathbb{1}}_{\mathrm{el}} \otimes (\hat{a}^{\dagger} + \hat{a})$. However, the relative magnitude of the electronic and photonic contributions in experiment is extremely reliant on the experimental setup (*e.g.*, cavity loss, direction of the probe, etc). Other works have used different quantities to explore the cross-correlation of various observables for the spectroscopic analysis of molecular systems in cavities.[6]

In experiment, usually the photonic contribution to the absorption and emission will dominate the intensity of the spectrum in Fabry–Pérot-type cavities.[7,8] However, for theoretical calculations, the excitonic absorption spectra (as well as other molecular properties), expressed here and in other works,[9–12] is better-suited to understand the effects of the cavity on the electronic sub-system and gives more direct insight into the *local* reactivity and *local* electronic reorganization in the molecule upon excitation of the polaritonic system. It is also important to recall that most of these reported results are in a single-mode cavity and, in principle, only represent the $\theta = 0$ special incident angle in a FP cavity.

Convergence test of the pQED approach

Fig. S1 shows convergence test for a benzaldehyde molecule coupled to an optical cavity. Here, the cavity is in resonance with the lowest, bright electronic transition, forming upper and lower polariton states. As functions of the number of Fock states (Fig. S1a) or adiabatic electronic states (Fig. S1b), the convergence of the upper (red) and lower (black) polariton energy is shown for two values of light-matter coupling strength A_0: 0.01 a.u. (solid,squares) and 0.02 a.u. (dotted, circles).

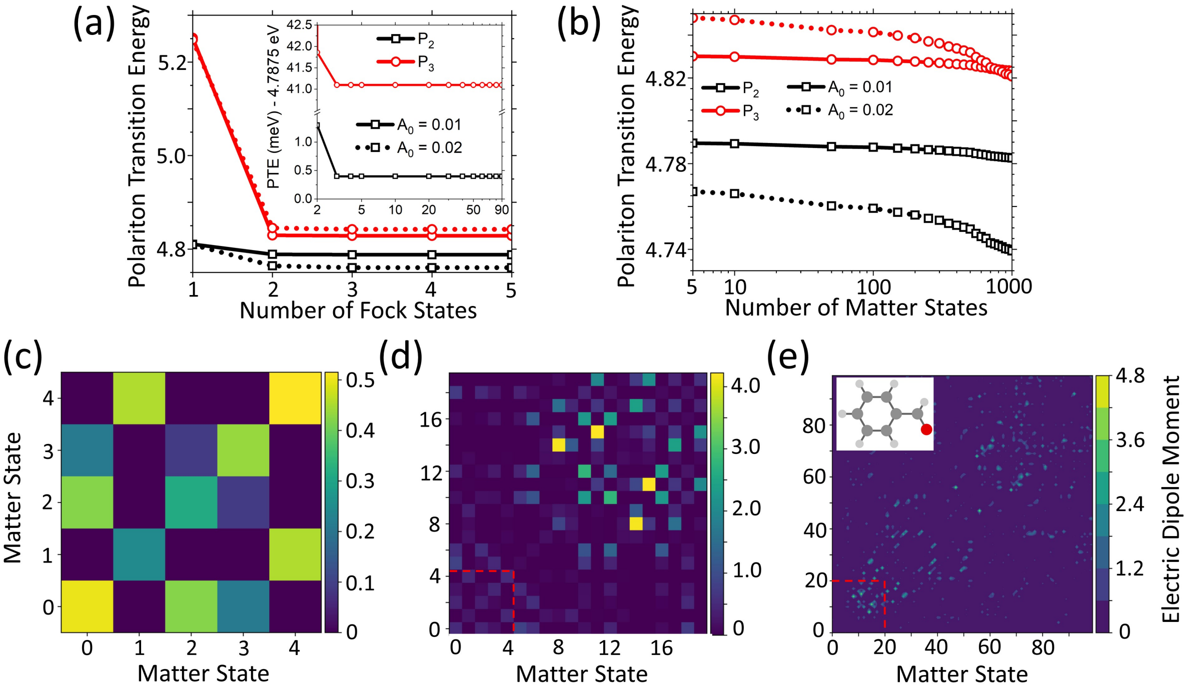

Figure S1: Convergence test of the benzaldehyde molecule (see inset in panel e) coupled to an optical cavity that is in resonant with the lowest bright singlet excitation of the bare molecular system. Similar tests have been performed in Ref. 9. (a) Convergence of the polaritonic eigenstates with respect to the number of Fock states and with with 50 electronic adiabatic states. Two molecule-cavity coupling strengths are shown: $A_0 = 0.01$ (solid line) and 0.02 (dashed line) a.u. for the upper polariton state Φ_3 (P_3, red) and lower polariton state Φ_2 (P_2, black). (b) Convergence of the polaritonic eigenstates with respect to the number of included electronic states, with 5 photonic Fock states. (c)-(e) The modulus of the bare molecular dipole matrix, $|\boldsymbol{\mu}_{\alpha\beta}|$, for the (c) 5, (d) 20, and (e) 100 lowest-energy electronic adiabatic states, computed with TD-DFT/ωB97XD/6-311G++. The color bar indicates the magnitude of the dipole.

Fig. S1a shows rapid convergence of the polariton energy as a function of the number of

photonic basis states used in the calculation (when using 50 electronic states). The energy converges to less than 1 meV using just 3 Fock states. The inset in Fig. S1 shows the convergence up to 90 Fock states, which changed by less than 1 meV compared to 5 Fock states. Fig. S1b shows the convergence with respect to the number of electronic states (using 5 Fock states). The convergence in electronic basis is much slower than for the photonic one due to the complexity of the molecular dipole matrix (Fig. S1c-e), which shows a change of less than 10 meV (30 meV) for a coupling strength of $A_0 = 0.01$ a.u. ($A_0 = 0.02$ a.u.) between 5 electronic states and 1000 electronic states. The slow convergence is primarily due to the dipole self-energy term in the Pauli-Fierz Hamiltonian (Eq. 1 in the main text), since it couples electronic states far-away in energy via the square of the molecular dipole matrix. Fig. S1c-e showcase the complexity of the dipole matrix at three levels: the first 5 electronic states, the first 20, and the first 100.

Similar convergence tests are also performed for all simulations presented in the main text. Details of the number of electronic adiabatic states and Fock states used for each calculation are provided in the Computational Details of the main text.

Additional Results of the Systems in the Main text

Fig. S2a shows the molecular dipole matrix for the formaldehyde molecule up to electronic state 20. Fig. S2b shows the magnitude of the avoided crossing as a function of the cavity frequency ω_c for a fixed light-matter coupling strength of $A_0 = 0.04$ a.u., showing a maxima near $\omega_c = 8.0$ eV. The large tunability of the avoided crossing with cavity frequency can be leveraged to modify non-adiabatic pathways in excited state reactions.

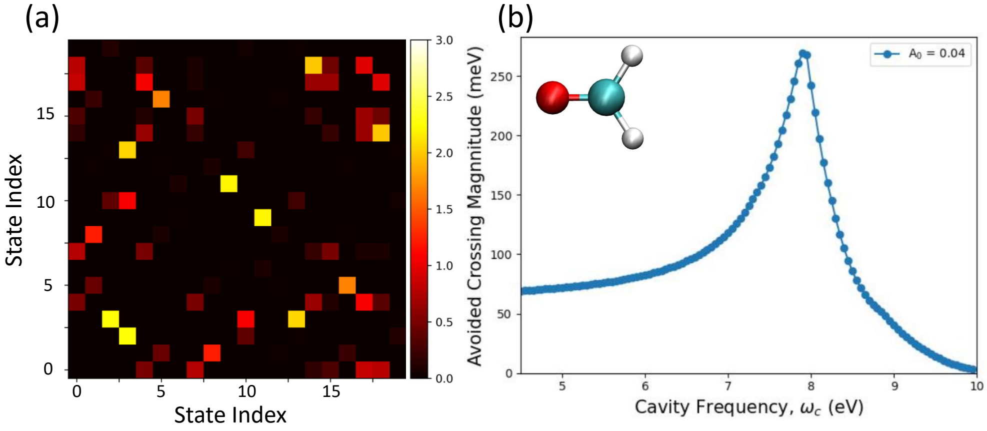

Figure S2: (a) Bare molecular transition dipole matrix of formaldehyde near the Frank-Condon point with a $R_{\mathrm{CO}} = 1.22$ Å. The color bar indicates the magnitude of the dipole matrix elements. (b) The magnitude of the avoided crossing $\mathcal{E}_{\mathrm{AC}} = \mathcal{E}_2(R_{\mathrm{CO}} = R_{\mathrm{AC}}) - \mathcal{E}_1(R_{\mathrm{CO}} = R_{\mathrm{AC}})$ as a function of cavity frequency ω_c with fixed coupling strength $A_0 = 0.04$ a.u., showing a maxima near $\omega_c = 8.0$ eV. Here, R_{AC}) indicates the avoid crossing point.

Fig. S3 shows the transition dipole matrices (up to 20 electronic states) for the aminopropenal molecule at the reactant (top), transition state (middle), and product (bottom) nuclear geometries in the X- (left) and Z-polarizations (right). These data show the complexity of the transition dipole matrix and thus the complexity of the dipole self-energy contribution ($\hat{H}_{\text{DSE}} \sim \hat{\mu}^2$).

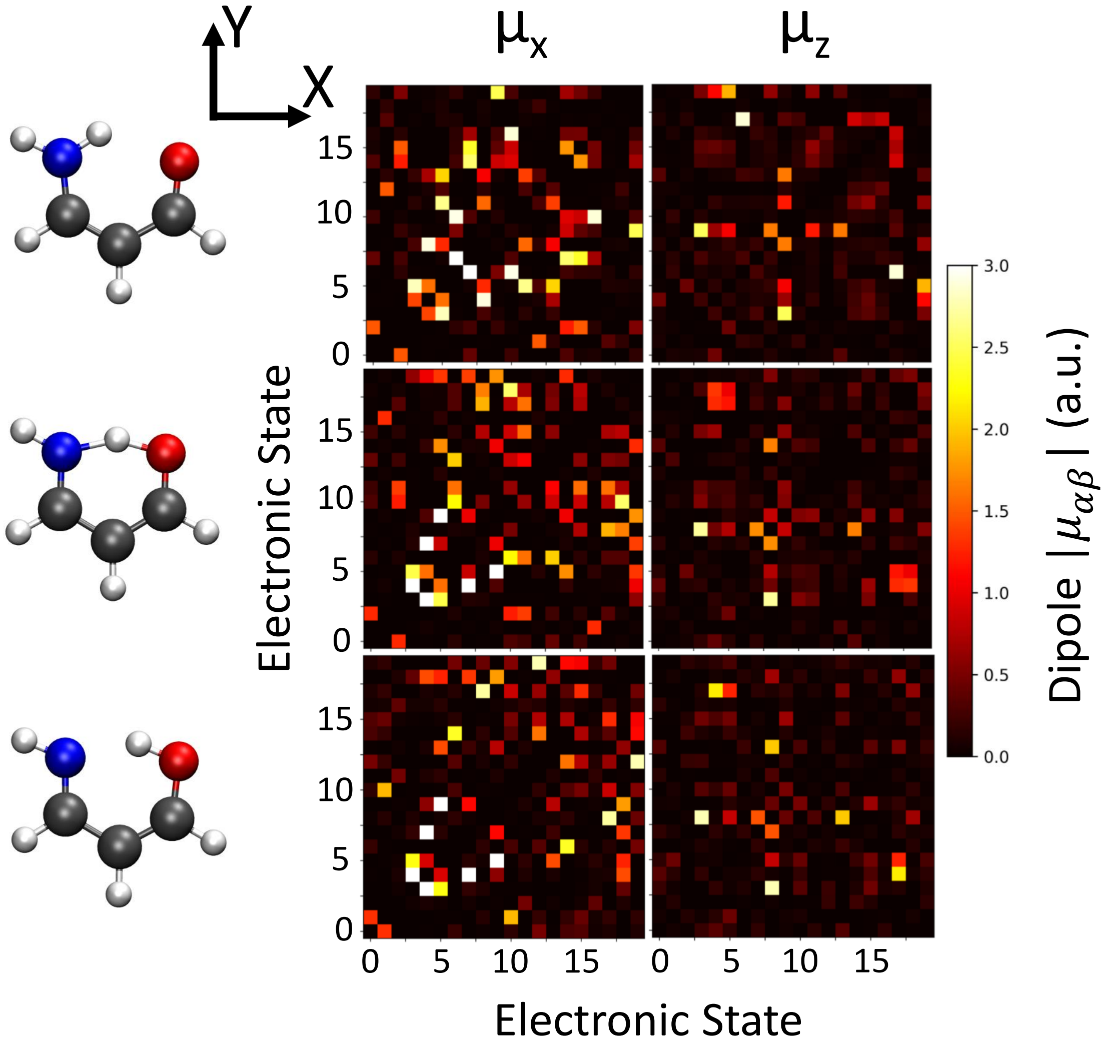

Figure S3: Bare molecular dipole matrices for the aminopropenal molecule projected along the x- (left) and z-directions (right) for the reactant (top), transition state (middle), and product (bottom) geometries. Note, in the main text, 500 molecular states were used but here only the 20 lowest-energy matrix elements are shown. The colorbar indicates the magnitude of the dipole in a.u.

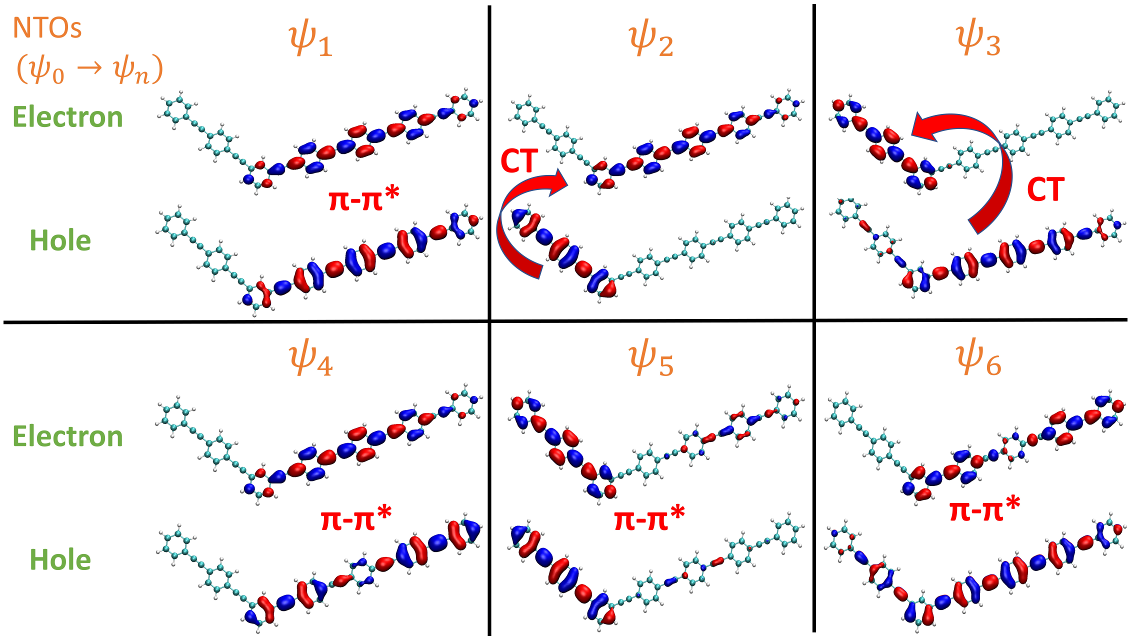

Figure S4: Dominant electronic natural transition orbitals for the 35PPE molecule outside of the cavity. The two charge-transfer states are the second and third electronic transitions and migrate charge in opposite directions, the lower-energy one transferring an electron from the short arm to the long arm. The first, fourth, fifth, and sixth transitions are mainly $\pi - \pi*$ on the long arm, with the exception of the fifth which is the $\pi - \pi*$ on the short arm.

Fig. S4 presents the natural transition orbitals for the bare 35 PPE molecule, providing an analysis of the various excited states present in the discussion of Fig. 3 in the main text. These characters provide the labeling for the Fig. 3a of the main text, such that "R" signifies right-localized electronic transition (excited states ψ_1, ψ_4, and ψ_6), "L" is left-localized (excited state ψ_5), and "CT" indicates a charge transfer excitation (excited states ψ_2 and ψ_3).

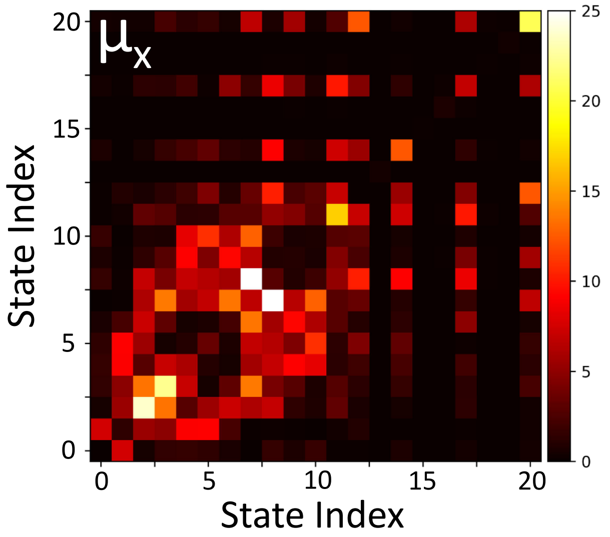

Figure S5: Transition dipole matrix for the lowest 20 electronic states in 35PPE projected in the X-direction. The value of the transition dipole is in a.u.

Transition dipole matrix for the lowest 20 electronic states in 35PPE projected in the X-direction, showing the complexity of the transition dipole matrix and thus the complexity of the dipole self-energy contribution ($\hat{H}_{\mathrm{DSE}} \sim \hat{\mu}^2$).

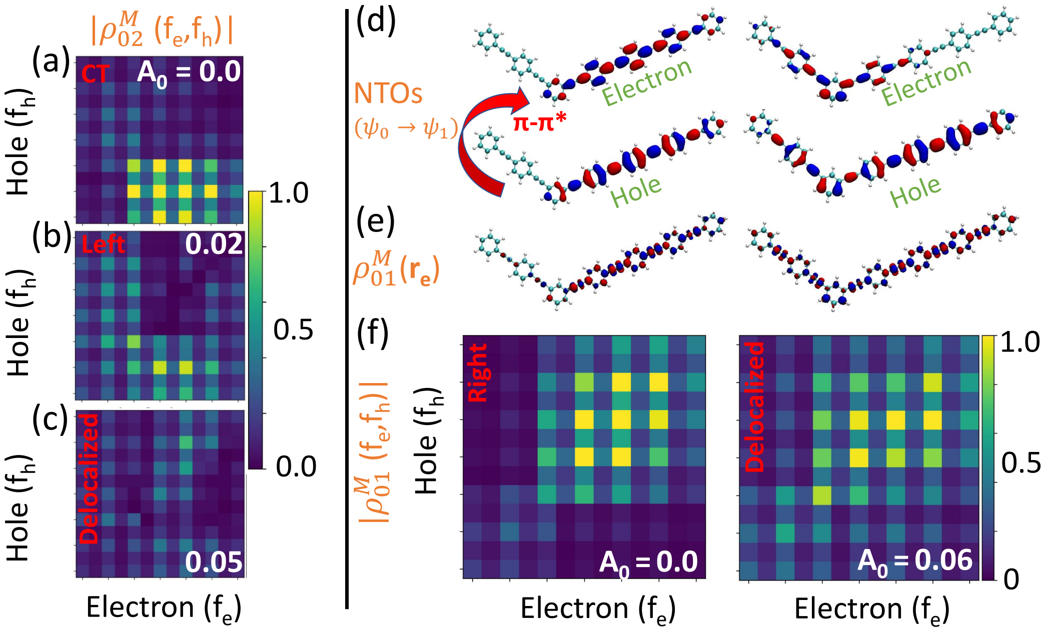

Figure S6: (a)-(c) show the real-space projected transition density matrices (see Eq. S13) for the three coupling strengths discussed in Fig. 3 of the main text for the same parameters. (d) Dominant natural transition orbitals for the electron and hole for the lowest-energy ground-to-excited polaritonic transition. The left and right columns correspond to coupling strengths of $A_0 = 0.0$ and 0.06 a.u., respectively. (e) Real-space projected polaritonic transition density. (f) Polaritonic transition density matrix.

Fig. S6a-c show the modulus of the real-space projected transition density matrices $|\rho_{02}^{\mathrm{M}}(f_{\mathrm{e}}, f_{\mathrm{h}})|$ (see definition in Eq. S13) for the three coupling strengths discussed in Fig. 3 of the main text for the same parameters. Here, the variables $\mathrm{f_e}$ and f_{h} represents the location of the electron and hole, respectively, in different fragments of the molecule. The electronic transition density matrix was constructed in the atomic orbital basis for a given electronic transition, and all atom-centered atomic orbitals were condensed to their atom and summed into 13 fragments (7 phenyl rings and 6 methylene bridges) from left to right along the 35PPE molecule, as implemented in Ref. 4. Using the transition density matrix, the spatial coherence of the polaritons during an excitation can now be directly analyzed.

The fragment transition density matrix showcases the CT nature of the second excited state at zero light-matter coupling $A_0 = 0.0$ a.u., since only *one* of the diagonal elements is largely populated while a large block on the lower right of the matrix is also populated.

Here, each row/column represents a fragment of the molecule from left to right (*e.g.*, alternating between a phenyl ring or a methylene bridge adding to 13 fragments). A large diagonal element in this matrix indicates that this fragment contributed largely to both the electron and hole reorganization upon excitation; whereas, a large off-diagonal element indicates charge reorganization between two fragments (*i.e.*, an electron starting in fragment f_e migrated to fragment f_h upon excitation). This matrix gives quantitative information regarding the spatial coherence between the electron and hole during excitation. Relating back to previous analysis, the NTOs are merely related to eigenvectors of this matrix, and the transition density is nothing more than the diagonal elements. At $A_0 = 0.02$ a.u., the transition density matrix becomes more delocalized between the lower right and upper left blocks but weaker in intensity while also populating the lower left diagonal elements. This indicates that the transition density will be largely residing on the left arm (which was noted above), but additionally, the weak coherence between the left and right arms is still present implying that the CT character has not completely disappeared.

Fig. S6d-f show the dominant NTOs, real-space projected transition density, and the transition density matrices for the lowest-lying polaritonic excited state at two coupling strengths: $A_0 = 0.0$ and 0.06 a.u. At $A_0 = 0.06$ a.u., the lowest-energy excited state becomes delocalized across both arms of the 35 PPE molecule since the character of the fifth bare electronic state becomes mixed into the first bare electronic state. See Fig. 3b,c in the main text for the polaritonic energies and absorption spectra as functions of light-matter coupling strength A_0.

Additional Analysis of Transition Density Analysis using Photonic Coordinate

Here, we use H_2 couples to cavity as a simple example to illustrate the polaritonic transition density matrix expressed in Eq. S10.

Fig. S7a depicts the polaritonic transition density $\rho_{0j}(\mathbf{r}_\mathrm{e}, q_\mathrm{c})$ for the four lowest-energy states of the light-matter hybrid system at zero coupling $A_0 = 0$ a.u. Note that all these states

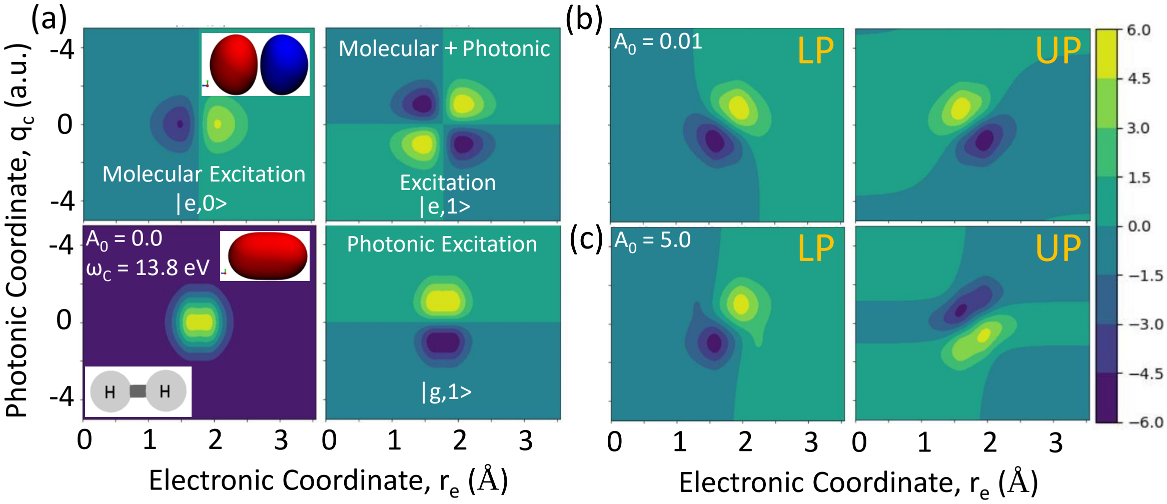

Figure S7: Transition density matrices $\xi_{0j}(r_e, q_c)$ for one electronic coordinate r_e and one photonic coordinate q_c of an H_2 molecule coupled to the cavity examining the for the ground state to the jth polaritonic state. The cavity frequency is in resonance with the lowest-energy molecular transition $\omega_c = 13.8$ eV. Panel (a) presents the ground state density $\xi_{00}(r_e, q_c)$ (lower left) and the three lowest-energy transition density matrices $\xi_{0j}(r_e, q_c)$ with $j \in \{1, 2, 3\}$ at zero coupling strength $A_0 = 0.0$ a.u.. The lower-left panel is the ground state total density (see isosurface of the bare molecular ground state density as an inset), the off-diagonal panels are composed of the molecular- or photonic-only excitations (soon to be called the lower and upper polaritons), and the upper-right panel is the high-energy double excitation of photon and matter DOFs. The ground-to-first excited transition density isosurface of the bare molecular system is shown as an inset for the upper left matrix in panel (a). Panel (b) shows the transition density matrices of the upper (right, UP) and lower (left, LP) polaritons at $A_0 = 0.01$ a.u., where now the transition density matrix shows “mixing” of the molecular and photonic DOFs. Panel (c) shows the transition density matrices for the UP and LP at an extreme coupling strength of $A_0 = 5.0$ a.u.

simply correspond to tensor product states of the electronic and photonic basis $|\psi_\alpha(\mathbf{R})\rangle \otimes |n\rangle$ (due to the zero light-matter coupling), such that they completely retain their original electronic character. The lower-left panel shows the polaritonic ground state ($|g,0\rangle$) density, the upper-left and lower-right panels show the ground-to-excited transition density for the single-excitation states $|e,0\rangle$ and $|g,1\rangle$, respectively, and the upper-right shows the transition density to the $|e,1\rangle$ state. The X-axis shows the 1D projection (along the H-H bond) of the real-space projected transition density, and the Y-axis is the photonic transition density along the photonic coordinate q_c in the single-mode cavity. The ground state of the photonic mode is exactly a Gaussian function, while the ground state of the molecule resembles that of two strongly overlapping Gaussians. The insets in the lower- and upper-left panels show the ground state density and real-space projected transition density for the molecular system, respectively.

Fig. S7b presents the photonic transition density $\rho_{0j}(\mathbf{r}_\mathrm{e}, q_\mathrm{c})$ (Eq. S10) for the transition from the ground state to the lower polariton state (left) and the transition from the ground state to the upper polaritons state (right), at a coupling strength of $A_0 = 0.01$ a.u. From these transition densities, one can observe an equal mixing of $|e,0\rangle$ and $|g,1\rangle$ states, which have degenerate energy at the resonant light-matter coupling. Fig. S7c present the same quantity at a larger coupling strength of $A_0 = 5.0$ a.u. The general shapes of these $\rho_{0j}(\mathbf{r}_\mathrm{e}, q_\mathrm{c})$ are similar compared to the low coupling strength in Fig. S7b. However, the phase of the upper polariton transition density has changed (while also becoming more stretched along the diagonal, indicating that mixing of between the resonant states $\{|g,1\rangle, |e,0\rangle\}$ and the high-energy state $|e,1\rangle$ is non-zero, even though the energy of $|e,1\rangle$ is twice that of the $\{|g,1\rangle, |e,0\rangle\}$ basis states. This mixing would be increasingly non-trivial if either the energy difference was smaller or the coupling was further increased. Although this method of analyzing the excited state polaritonic transitions is rather simplified due to the model (*i,e.*, H_2), it has the potential to provide a fundamental view into more complicated situations where there is one “important” dimension of the matter and where mixing of multiple Fock or

matter excitations can be uniquely visualized. Two examples of such cases are (I) low-energy cavities such that multiple Fock states appear below the onset of molecular transitions[13] and (II) multi-mode cavities such as the "quasi-continuous" parallel modes in the Fabry-Perot[14] or plasmonic nanoparticle[15] designs. The transition density between states of mixed Fock state or mixed cavity mode character may give a unique insight into the correlations between these DOFs.

References

(1) Tretiak, S.; Mukamel, S. Density Matrix Analysis and Simulation of Electronic Excitations in Conjugated and Aggregated Molecules. *Chem. Rev.* **2002**, *102*, 3171–3212.

(2) Plasser, F.; Bäppler, S. A.; Wormit, M.; Dreuw, A. New tools for the systematic analysis and visualization of electronic excitations. II. Applications. *J. Chem. Phys.* **2014**, *141*, 024107.

(3) Plasser, F.; Wormit, M.; Dreuw, A. New tools for the systematic analysis and visualization of electronic excitations. I. Formalism. *J. Chem. Phys.* **2014**, *141*, 024106.

(4) Lu, T.; Chen, F. Multiwfn: A multifunctional wavefunction analyzer. *J. Comput. Chem.* **2012**, *33*, 580–592.

(5) Martin, R. L. Natural transition orbitals. *J. Chem. Phys.* **2003**, *118*, 4775–4777.

(6) Ruggenthaler, M.; Tancogne-Dejean, N.; Flick, J.; Appel, H.; Rubio, A. From a quantum-electrodynamical light–matter description to novel spectroscopies. *Nat Rev Chem* **2018**, *2*, 1–16.

(7) Engelhardt, G.; Cao, J. Unusual dynamical properties of disordered polaritons in microcavities. *Phys. Rev. B* **2022**, *105*, 064205.

(8) Herrera, F.; Spano, F. C. Absorption and photoluminescence in organic cavity QED. *Phys. Rev. A* **2017**, *95*, 053867.

(9) Yang, J.; Ou, Q.; Pei, Z.; Wang, H.; Weng, B.; Shuai, Z.; Mullen, K.; Shao, Y. Quantum-electrodynamical time-dependent density functional theory within Gaussian atomic basis. *J. Chem. Phys.* **2021**, *155*, 064107.

(10) Flick, J.; Narang, P. Ab initio polaritonic potential-energy surfaces for excited-state nanophotonics and polaritonic chemistry. *J. Chem. Phys.* **2020**, *153*, 094116.

(11) Wang, D. S.; Yelin, S. F.; Flick, J. Defect Polaritons from First Principles. *ACS Nano* **2021**, *15*, 15142–15152.

(12) Flick, J.; Welakuh, D. M.; Ruggenthaler, M.; Appel, H.; Rubio, A. Light–Matter Response in Nonrelativistic Quantum Electrodynamics. *ACS Photonics* **2019**, *6*, 2757–2778.

(13) Mandal, A.; Montillo Vega, S.; Huo, P. Polarized Fock States and the Dynamical Casimir Effect in Molecular Cavity Quantum Electrodynamics. *J. Phys. Chem. Lett.* **2020**, *11*, 9215–9223.

(14) Qiu, L.; Mandal, A.; Morshed, O.; Meidenbauer, M. T.; Girten, W.; Huo, P.; Vamivakas, A. N.; Krauss, T. D. Molecular Polaritons Generated from Strong Coupling between CdSe Nanoplatelets and a Dielectric Optical Cavity. *J. Phys. Chem. Lett.* **2021**, *12*, 5030–5038.

(15) Sample, A. D.; Guan, J.; Hu, J.; Reese, T.; Cherqui, C. R.; Park, J.-E.; Freire-Fernández, F.; Schaller, R. D.; Schatz, G. C.; Odom, T. W. Strong Coupling Between Plasmons and Molecular Excitons in Metal–Organic Frameworks. *Nano Lett.* **2021**, *21*, 7775–7780.
