# Supplementary material for: Investigating Molecular Exciton Polaritons Using Ab Initio Cavity Quantum Electrodynamics
Source: J Phys Chem Lett. 2023 Jun 21;14(25):5901–13. doi: 10.1021/acs.jpclett.3c01294 (PMC10316409; doi:10.1021/acs.jpclett.3c01294)
Supplement: Supplementary file 2 — jz3c01294_si_002.pdf [file jz3c01294_si_002.pdf]

jz-2023-01294y.R1

Name: Peer Review Information for "Investigating Molecular Exciton-Polaritons Using ab Initio Cavity Quantum Electrodynamics"

First Round of Reviewer Comments

Reviewer: 1

Comments to the Author

1. What is the major advance reported in the paper?

The authors compare the parameterized QED (pQED) approach with the self-consistent QED (scQED) results that have been recently investigated, including molecular polaritons, cavity-mediated proton-transfer reaction, conjugated polymers, and single-walled carbon nanotube. The advantage of the pQED approach is that the molecule-cavity interactions are treated exactly, without being restricted by the level of approximation used for treating electronic correlation. Furthermore, the pQED approach utilizes existing ab initio electronic structure calculations and does not need the development of new DFT functionals as in the scQED scheme. These advantages make the pQED approach a promising method for calculating properties related to molecular exciton-polaritons.

2. What is the immediate significance of this advance?

The advance highlights that the pQED approach can achieve accurate results consistent with the scQED approach. However, the authors admit that “the scQED schemes may require substantially less computational effort than the analogous pQED scheme.” That being said, considering that the pQED scheme incorporates the exact electron-photon interaction, it is worthwhile to explore the potential of pQED. In general, the manuscript holds great interest for the polariton community and carries immediate significance.

3. Technical suggestions

Overall, the manuscript is well written and clear. There are a few parts that I find confusing:

a. On Page 3, I am uncertain about the following sentence: "“Although, it should be noted that including a many-level generalization, including DSE and highly oscillatory terms (i.e., not making the rotating wave approximation) as is done in the full PF Hamiltonian causes this direct analogy to be weak as more complex effects arise at large coupling strengths where non-linear effects are ubiquitous.”

b. In Figure 2 (c), it is surprising that the pQED-DFT outperform scQED-DFT when calculating the transition state energy. The authors argue that: “The higher accuracy achieved by pQED approach is likely due to the exact electron-photon correlation provided by the direct diagonalization of the exact interaction term used in Eq. 1.” However, it is unclear why the pQED-DFT does not exhibit the same level of accuracy when calculating the product state energy.

c. In Figure 3, the results of the NTO provide an explanation for the characteristics observed in the transition density. At a larger light-matter coupling strength, it shows that the electron distribution is delocalized, whereas the hole distribution is only weakly delocalized. However, it is unclear about the specific factors that differentiate between electrons and holes.

d. Some notations are not consistent:

i. In Eq. (3), is the the total dipole operator (mu) a 3D vector, and should it be indicated in bold?

ii. The subscript R should be indicated in bold for the nuclear kinetic energy operator (T_R) on line 53 and 55 of Page 3.

Reviewer: 2

Comments to the Author

Weight et al. present a technique to compute molecular polaritons by extracting the necessary quantities form TD-DFT calculations. The paper is well written and presents a comparison of the presented technique with fully self consistent QED-SCF methods. The topic is timely and the authors demonstrate that this technique seems to be suitable for addressing molecular polaritonic questions.

However, I am bit surprised that it has been submitted as letter. For a letter it seems to be too technical and a bit too long in total. JPCLs requirement - "Letters are short articles that should present significant new physical insights and/or extremely important results" - does not seem to be fulfilled here, as I do not think that it presents "significant new physical insights", but rather a computational technique. As such it would fit better into, for example, JCTC.

I also find the title of the paper somewhat misleading. The authors call it "parameterized QED (pQED) approach", but sell it as "ab initio QED" in the title, which seems like a contradiction. Since their technique is not self consistent and uses TD-DFT to obtain energies and transition dipole moments, the expression "ab initio QED" is perhaps a bit of a stretch.

The authors use the Pauli-Fierz Hamiltonian. In its formulation in photon displacement coordinates it requires that the zero-field condition is fulfilled, which becomes important as soon as the molecule has a non-zero dipole moment. Self consistent electronic structure methods as presented, for example in Ref 46 or Haugland et al. PRX, 10, 041043 (2020), this is taken into account via mu'= mu-<mu>. From the formulation in this paper it is not clear to me where

this enters here.

The authors claim that Fig 2c shows that their approach "outperforms" scQED-DFT. However, it is not discussed what causes this discrepancy. It just assumed here that scQED-DFT is the more accurate method without further testing.

Author's Response to Peer Review Comments:

##
The Reviewer comments are shown in **black**, author comments are shown in **blue**, and changes to the manuscript are shown in **red**.
##

Reviewer: 1

Recommendation: This paper is publishable subject to minor revisions noted. Further review is not needed.

We thank the Reviewer #1 for their positive recommendation of our work. Please find our point-by-point response below.

1. What is the major advance reported in the paper? The authors compare the parameterized QED (pQED) approach with the self-consistent QED (scQED) results that have been recently investigated, including molecular polaritons, cavity-mediated proton-transfer reaction, conjugated polymers, and single-walled carbon nanotube. The advantage of the pQED approach is that the molecule-cavity interactions are treated exactly, without being restricted by the level of approximation used for treating electronic correlation. Furthermore, the pQED approach utilizes existing ab initio electronic structure calculations and does not need the development of new DFT functionals as in the scQED scheme. These advantages make the pQED approach a promising method for calculating properties related to molecular exciton-polaritons.

2. What is the immediate significance of this advance? The advance highlights that the pQED approach can achieve accurate results consistent with the scQED approach. However, the authors admit that “the scQED schemes may require substantially less computational effort than the analogous pQED scheme.” That being said, considering that the pQED scheme incorporates the exact electron-photon interaction, it is worthwhile to explore the potential of pQED. In general, the manuscript holds great interest for the polariton community and carries immediate significance.

In extension to the Reviewer #1’s overview of the major advance and immediate significance of our work (Comments 1 and 2), we believe the timely publication of this work warrants consideration since the majority of the electronic structure community does not have immediate and user-friendly access to these reconstructed scQED methods. Our opinion is that the pQED approach will allow the broad community of electronic structure and its ubiquitous applications to immediately and simply extend already-developed to the computation of polaritonic properties without the need to install new packages or learn new techniques.

3. Technical suggestions Overall, the manuscript is well written and clear. There are a few parts that I find confusing:

a. On Page 3, I am uncertain about the following sentence: "“Although, it should be noted that including a many-level generalization, including DSE and highly oscillatory terms (i.e., not making the rotating wave approximation) as is done in the full PF Hamiltonian causes this direct analogy to be weak as more complex effects arise at large coupling strengths where non-linear effects are ubiquitous.”

Response: We agree with the Reviewer #1 the wording of this sentence was confusing. We have removed this confusing and unnecessary statement.

b. In Figure 2 (c), it is surprising that the pQED-DFT outperform scQED-DFT when calculating the transition state energy. The authors argue that: “The higher accuracy achieved by pQED approach is likely due to the exact electron-photon correlation provided by the direct diagonalization of the exact interaction term used in Eq. 1.” However, it is unclear why the pQED-DFT does not exhibit the same level of accuracy when calculating the product state energy.

Response: To be clear, pQED not only outperforms the scQED-DFT in the transition energy calculations, it also outperforms the scQED-DFT in terms of the product energy calculations. It is just that for the product energy, the pQED is less accurate when comparing the benchmark results of the scQED-CCSD.

As we claimed, it is not surprising that pQED-DFT outperforms scQED-DFT, for both the transition energy and product energy calculations. The possible reason, as we have suggested, is due to the exact electron-photon correlation used in pQED (in the Hamiltonian) and the superior treatment of the electron-electron correlation (with the wB97XD hybrid functional). In the direct diagonalization procedure in pQED, the only quantity needed for the interactions is the electron-photon interaction term in the Hamiltonian, which is exact. In the scQED-DFT approach, one needs to provide the electron-photon correlation functional term for the single particle description, as well as one for the electron-electron correlation. The approximation one has to make for such exchange-correlation functionals ultimately limits the accuracy of scQED. With that said, one can certainly improve the accuracy of scQED-DFT by deriving more accurate exchange-correlation correlation functionals.

We have added a concluding sentence to right column on page 7 to directly point out this overall result between the two approaches, which reads as, “Overall, the pQED-TD-DFT/ωB97XD approach outperforms the scQED-DFT/OEP in both the transition state and product geometries compared to the benchmark scQED-CCSD results.”.

Further, we have more clearly pointed out the reasons why pQED-TDDFT outperforms scQED-DFT in both cases by amending the final paragraph on page 7 as, "The higher accuracy achieved by pQED approach is likely due to two primary effects: (I) the exact electron-photon correlation provided by the direct diagonalization of the exact interaction term used in Eq.~\ref{EQ:PF_HAM} as well as (II) the heightened level of theory for the bare electron-electron correlations due to the more accurate ωB97XD hybrid functional."

c. In Figure 3, the results of the NTO provide an explanation for the characteristics observed in the transition density. At a larger light-matter coupling strength, it shows that the electron distribution is delocalized, whereas the hole distribution is only weakly delocalized. However, it is unclear about the specific factors that differentiate between electrons and holes.

Response: We thank the Reviewer #1 for this insightful comment. We will try to address the overall reason for why there are asymmetric modifications to the electron and hole distributions.

Excitations in the molecular system (at least at the linear response TD-DFT level) are composed of linear combinations of excited electrons (in previously unoccupied single-particle orbitals) and excited holes (in previously fully filled occupied orbitals – in the spin-restricted sense). The excitations can be thought of as these excited electron/hole pairs, and the high-frequency excitations sample single-particle orbitals farther from the highest occupied (HOMO) and lowest unoccupied (LUMO) molecular orbitals. Natural transition orbitals (NTOs) are a simple way to take these linear combinations of electron/hole excitations and condense them into a compact basis for visualization. In this way, the NTO for the electron should be a good representation for its distribution (including its phase information), and likewise for the hole. This representation is more akin to the chemist-style single-particle orbital representation in the ground state and thus offers a nice way to visualize these localization properties and subsequent charge-transfer characteristics inside the cavity.

Practically speaking, for the cavity to induce large changes to the electron's NTO distribution and not the hole's, the cavity-induced changes to electronic states would need to mix (or cause crossings between) excited states that share a common dominant virtual orbital in its expansion. For example, if there was an excitation primarily composed of the j->a single-particle transition (\textit{i.e.}, in a singly excited Slater determinant sense) and another, j->b. Since the two excitations share the same occupied orbital (i.e., j), the cavity-induced mixing would primarily affect the excited electron distribution (since a is not equal to b) and not the hole distribution. We expect this to be the reason for the asymmetric changes to the electron and hole NTOs present in Figure 3 of the current work. However, the investigation of the

single-particle orbitals and their role in the transition is beyond the scope of this work and will be the subject of future work.

While we did not include this discussion here, other works have noted the asymmetric modification to the single-particle orbitals in scQED approaches (notably scQED-HF in Ref. [1]). Here, the authors of Ref. [1] note that the virtual/unoccupied orbitals are more strongly affected than the occupied ones. The authors mention, “Both occupied and unoccupied orbitals are affected by the cavity parameters, however, larger changes are typically observed for the latter.” To my knowledge, this trend has not been studied or yet well-explained.

[1] Riso et al., Molecular orbital theory in cavity QED environments, Nature Commun. 13, 1368 (2022), https://www.nature.com/articles/s41467-022-29003-2

We have added a paragraph regarding the observation of asymmetric changes to the electron and hole distributions in the left column of page 9 (onto the beginning of page 10) as, “As noted above, the NTOs for the electron and hole distributions for the 35PPE molecule exhibit asymmetric changes such that the electronic NTO was largely modified, while the hole NTO was only weakly modified. The presence of asymmetric modifications implies an asymmetry of affected occupied and virtual orbitals that comprise the various bare molecular excitations. Practically speaking, for the cavity to induce large changes to the electron's NTO distribution and not the hole's, the cavity-induced changes to electronic states would need to mix (or cause crossings between) excited states that share a common dominant virtual orbital in its expansion. For example, if there was an excitation primarily composed of the $j\rightarrow a$ single-particle transition (\textit{i.e.}, in a singly excited Slater determinant sense) and another, $j\rightarrow b$. Since the two excitations share the same occupied orbital (\textit{i.e.}, j), the cavity-induced mixing would primarily affect the excited electron distribution (since $a \ne b$) and not the hole distribution. We expect this to be the reason for the asymmetric changes to the electron and hole NTOs present in Fig. 3. Such an asymmetry in the cavity-induced changes to molecular properties (in occupied/virtual orbitals in Ref.~\citenum{riso_molecular_2022} or electron/hole NTOs in the current work) has been pointed out in previous work.\cite{riso_molecular_2022} However, the investigation of the single-particle orbitals and their role in the transition is beyond the scope of this work and will be the subject of future work.”

d. Some notations are not consistent:
i. In Eq. (3), is the total dipole operator (mu) a 3D vector, and should it be indicated in bold?

We thank the Reviewer #1 for pointing this out. We have bolded the molecular dipole where needed throughout the manuscript.

ii. The subscript R should be indicated in bold for the nuclear kinetic energy operator (T_R) on line 53 and 55 of Page 3

We thank the Reviewer #1 for pointing this out. We have bolded the molecular positions where needed throughout the manuscript.

Reviewer: 2

Recommendation: While the work is good and publishable, a more appropriate journal is recommended such as JCTC

Comment 1: Weight et al. present a technique to compute molecular polaritons by extracting the necessary quantities form TD-DFT calculations. The paper is well written and presents a comparison of the presented technique with fully self consistent QED-SCF methods. The topic is timely and the authors demonstrate that this technique seems to be suitable for addressing molecular polaritonic questions. However, I am bit surprised that it has been submitted as letter. For a letter it seems to be too technical and a bit too long in total. JPCLs requirement - "Letters are short articles that should present significant new physical insights and/or extremely important results" - does not seem to be fulfilled here, as I do not think that it presents "significant new physical insights", but rather a computational technique. As such it would fit better into, for example, JCTC.

Response: We thank the Reviewer #2 for their positive evaluation of our work. While we agree that the current work provides a framework for computing polaritonic states and their subsequent properties, we believe that the selected applications of the technique (Figures 3 and 4) demonstrate the usefulness of the approach to a broad range of applications such as light-harvesting (Figure 3 – which investigates cavity-modulated charge transfer states) and light-emitting (Figure 4 – which explores the expected cavity-induced changes to the photoluminescence properties of single-walled carbon nanotubes), and indeed provide "new physical insights and/or extremely important results", such as how to use light-matter interactions to modulated charge transfer properties in molecules.

Further, we believe the timely publication of this work warrants consideration since the majority of the electronic structure community does not have immediate and user-friendly access to these reconstructed QED-SCF methods. Our opinion is that the pQED approach will allow the broad community of electronic structure and its ubiquitous applications to immediately and simply extend already-developed tools to the computation of polaritonic properties without the need to install new packages or learn new techniques.

Please find our point-by-point responses below to all reviewer comments.

Comment 2: I also find the title of the paper somewhat misleading. The authors call it "parameterized QED (pQED) approach", but sell it as "ab initio QED" in the title, which seems like a contradiction. Since their technique is not self consistent and uses TD-DFT to obtain energies and transition dipole moments, the expression "ab initio QED" is perhaps a bit of a stretch.

Response: Our opinion is that "*ab initio* QED" is a broad category which encompasses both self-consistent QED (scQED) methods as well as the parameterized QED (pQED) approach (which still relies on the self-consistent evaluation of the bare electron-electron interactions). The "*ab initio*" component is the realistic nature of the molecular DOFs where *ab initio* electronic structure calculations are required. In this sense, both scQED and pQED are *ab initio* approaches. The photonic component does not detract from this since the photonic DOFs and their interactions with the molecular DOFs are treated exactly by direct diagonalization of the Hamiltonian, which, in our opinion, should also be considered an *ab initio* approach.

We thus respectfully request to keep the original title of the manuscript to provide a more general language for the reader.

Comment 3. The authors use the Pauli-Fierz Hamiltonian. In its formulation in photon displacement coordinates it requires that the zero-field condition is fulfilled, which becomes important as soon as the molecule has a non-zero dipole moment. Self consistent electronic structure methods as presented, for example in Ref 46 or Haugland et al. PRX, 10, 041043 (2020), this is taken into account via mu'= mu-<mu>. From the formulation in this paper it is not clear to me where this enters here.

Response: In this work, we are using the vacuum Fock states, which was referred to by the Reviewer #2 as the "zero field condition", meaning the Fock states are centered around 0 value for the photonic coordinate. When approaching the complete basis limit, the Fock states will indeed converge the results. For all numerical calculations in this work, we found 5-10 Fock states are enough to converge the results. An alternative basis, such as polarized Fock states (Ref. [2] below) or the similar strategy in Ref. [1] below, are potentially better due to the shift of the Fock state that can facilitate faster convergence in the photonic basis.

Additional Discussion and Changes to the Manuscript
In the language of photonic coordinates, the Pauli-Fierz Hamiltonian can be interpreted as a photonic shift by a term proportional to the molecular dipole operator and the light-matter coupling strength. In this sense, one can shift away the direct coupling term by choosing a basis of shifted (and electronic-state specific) Fock states for the photonic DOFs. Many works in scQED approaches use this shifted basis, often referred to as the generalized coherent state (GCS) basis[1] and very related to the polarized Fock state (PFS) basis.[2] In

general, such an entangled basis for the photonic DOFs should produce a more rapidly converging result in terms of the number of included basis states; however, we find that for realistic systems, on a small number of vacuum Fock states (~5-10) for the photonic basis are required to achieve a reasonable accuracy for moderate light-matter coupling strengths. Our results, and the results of others,[3] suggest that the complication relies primarily on the truncation of the electronic states, which converge slowly with the number of included basis states.

To be clear, in the current work, we use the unperturbed vacuum Fock states as the basis for the photonic DOFs, which do not depend on the molecular dipole operator. Therefore, the coherence state shift, which is proportional to the expectation value of the molecular dipole operator <μ>, does not appear in our Hamiltonian. As expected, the choice of basis does not, and should not, affect the resulting observables for the polaritonic states.

[1] Haugland et al. PRX, 10, 041043 (2020)
[2] Mandal et al., J. Phys. Chem. Lett. 2020, 11, 21, 9215–9223
[3] Yang et al., J. Chem. Phys. 155, 064107 (2021)

We have added a new paragraph to the first column on page 4 to address this confusion and draw a direct connection to the literature.

“It is worth noting that in the community of ab initio QED for realistic molecular systems, often a coherent states transformation is performed on the PF Hamiltonian (Eq. 1). This unitary-transformed Hamiltonian can be written in terms of the fluctuations in the dipole operator $\Delta \hat{\mu} = \hat{\mu} - \langle \hat{\mu} \rangle$ and its square $(\Delta \hat{\mu})^2$. In this sense, one can shift away the direct coupling term by choosing a basis of shifted (and electronic-state specific) Fock states for the photonic DOFs. Many works in scQED approaches use this shifted basis, often referred to as the generalized coherent state (GCS) basis~\cite{haugland_coupled_2020,philbin_generalized_2014 } and very related to the polarized Fock state (PFS) basis.\cite{mandal_polarized_2020,mandal_theory_2023} The GCS basis is convenient for self-consistent methods, since the expectation value of the dipole operator can be evaluated at each self-consistent cycle and to achieve the optimal photonic basis at each iteration. The photonic basis states can be interpreted as coherent states (\textit{i.e.}, linear combinations of vacuum Fock states), which, in general, are expected to provide a more rapidly converging basis. However, in this letter, we use the unperturbed vacuum Fock states for simplicity, since the primary complication resides in the truncation of the electronic basis rather than the photonic one for realistic, ab initio systems.”

Comment 4. The authors claim that Fig 2c shows that their approach "outperforms" scQED-DFT. However, it is not discussed what causes this discrepancy. It just assumed here that scQED-DFT is the more accurate method without further testing.

Response: Figure 2c explores the quantitative comparison between the pQED-TDDFT approach (current work, red in Figure 2c) and that of two opposing scQED approaches, one of density functional theory (DFT, green in Figure 2c) using the newly developed optimized effective potential (OEP) functional and one of coupled cluster singles doubles (CCSD, black in Figure 2c). We take the scQED-CCSD result as the benchmark for our pQED approach, since the level of correlation between electron, photonic, and combined electron-photon DOFs is substantially increased compared to DFT or TD-DFT.

Our results (Figure 3c) showcases that the pQED-TDDFT (current work) outperforms the scQED-DFT in both the transition state and product geometries, since the pQED-TDDFT results align more closely with the scQED-CC results. In fact, for the transition state geometry, the pQED and scQED-CCSD results are within 30 meV, which is well within the expectations between DFT and CCSD outside the cavity.

The relative failure of the scQED-DFT/OEP approach can be explained by a few things. The OEP functional, as is the case with many scQED approaches, treats the electronic correlation at the same or similar level as the photonic and the combined electron-photonic correlation, which turns out to be similar to a local density approximation (LDA) for the correlation. In the current work, the electronic correlation was treated using the wB97XD hybrid exchange-correlation functional, which is known to provide accurate, high-level results for bare molecular systems. In addition to this, the electron-photon interactions are, in principle, treated exactly through the exact diagonalization approach in the pQED scheme. This alleviates any approximations between the electron-photon interactions up to the truncation of the basis sets, for both the electronic and photonic DOFs.

To address this concern or possible confusion, we have added a concluding sentence to the last paragraph on page 7 (continued on page 8) to directly point out this overall result between the two approaches, which reads as, "Overall, the pQED-TD-DFT/ωB97XD approach outperforms the scQED-DFT/OEP in both the transition state and product geometries compared to the benchmark scQED-CCSD results.". We also modified the discussion of the scQED-DFT/OEP results as, "The higher accuracy achieved by pQED approach is likely due to two primary effects: (I) the exact electron-photon correlation provided by the direct diagonalization of the exact interaction term used in Eq.~\ref{EQ:PF_HAM} as well as (II) the heightened level of theory for the bare electron-electron correlations due to the more accurate ωB97XD hybrid functional."

The Reviewer #2's comment "It just assumed here that scQED-DFT is the more accurate method without further testing." was not clear. We claim that the scQED-DFT was the least accurate method. Possibly, the Reviewer #2 meant to write "pQED-TDDFT" instead of "scQED-DFT". In the current work, Figures 1 and 2 were benchmark studies whose molecules had

been previously explored using various scQED approaches. In both cases, the pQED approach of the current work was able to reproduce the quantitative features of both systems resulting from the formation of polaritonic states.
